# Switching Spike Plasticity Shapes ACE2 Engagement Across SARS‐CoV‐2 Variants

**DOI:** 10.1002/advs.77290

**Published:** 2026-08-31

**Authors:** Sarah Stainer, Rong Zhu, Manuel Reithofer, Gerhard Stadlmayr, Katharina Stadlbauer, Panchika Prangkio, Christoph Rippel, Markus Hahn, Aniko Eva Kasztner, Stefanie Stohl, Petr Bednar, Daniel Ruzek, David Glück, Robert Tampé, Gordana Wozniak‐Knopp, Yoo Jin Oh, Miriam Klausberger, Peter Hinterdorfer

**Affiliations:** ^1^ Institute of Biophysics Johannes Kepler University Linz Linz Austria; ^2^ Department of Biotechnology and Food Science, Institute of Molecular Biotechnology BOKU University Vienna Austria; ^3^ Department of Chemistry Faculty of Science Chiang Mai University Chiang Mai Thailand; ^4^ Veterinary Research Institute Brno Czech Republic; ^5^ Department of Experimental Biology Faculty of Science Masaryk University Brno Czech Republic; ^6^ Faculty of Science University of South Bohemia Ceske Budejovice Czech Republic; ^7^ Institute of Parasitology Biology Centre of the Czech Academy of Sciences Ceske Budejovice Czech Republic; ^8^ Institute of Biochemistry Biocenter Goethe University Frankfurt Frankfurt Germany

**Keywords:** atomic force microscopy, biophysics, evolution, force spectroscopy, glycosylation, mass spectrometry, protein engineering, protein purification, severe acute respiratory syndrome coronavirus 2 (SARS‐CoV‐2), virology

## Abstract

Conformational plasticity allows class I viral fusion proteins, including the SARS‐CoV‐2 spike, to undergo major structural rearrangements that support receptor binding and membrane fusion, making them key vaccine and antiviral targets. Using high‐speed atomic force microscopy (AFM) and single‐molecule force spectroscopy, we analyzed the ancestral and nine spike variants and found an evolutionary trend from increased flexibility in early strains to greater rigidification beginning with Delta, fluctuating plasticity in Omicron sublineages, and pronounced compaction in JN.1. Delta favored multivalent ACE2 engagement through its three receptor‐binding domains, whereas later Omicron variants shifted toward predominantly single‐site interactions. This change was compensated by stronger RBD–ACE2 bond stability. Overall, spike evolution appears to balance openness and compaction to regulate receptor accessibility, mobility, and immune escape while preserving or enhancing entry efficiency. These single‐molecule measurements define functional constraints on viral adaptation and may inform prediction of future variants and design of improved countermeasures.

## Graphical abstract

Conformational plasticity governs SARS‐CoV‐2 spike function and ACE2 recognition. Using high‐speed AFM and single‐molecule force spectroscopy, we analyzed the ancestral spike and nine variants, revealing progressive rigidification from Delta, variable plasticity in Omicron sublineages, and JN.1 compaction. Concurrently, ACE2 binding shifted from multivalent to fewer, stronger interactions, optimizing receptor engagement, immune escape, and viral entry.

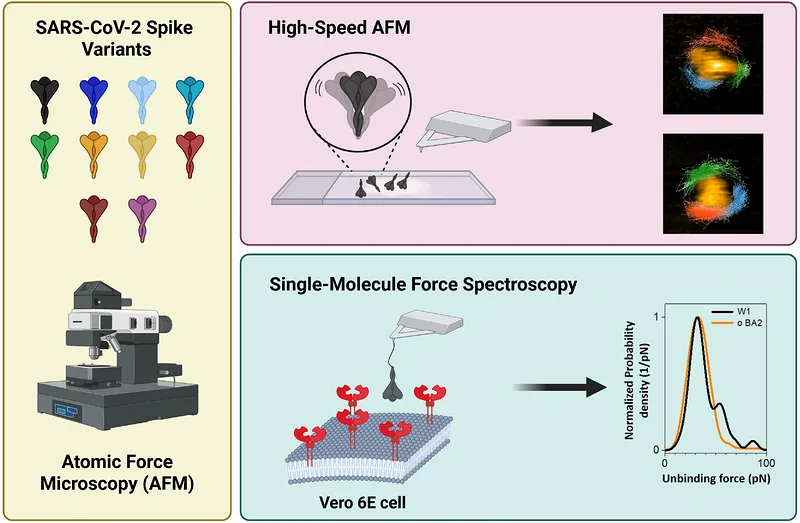

## 1 Introduction

Conformational plasticity is an important trait of viral binding and fusion proteins as it enables drastic structural rearrangements to exert distinct functions during viral entry. As the viral entry pathway is frequently the preferred target of prophylactic and therapeutic countermeasures, viral surface proteins enabling viral attachment and/or penetration are key components of current vaccines and targets of antivirals [1, 2]. Consequently, much of the adaptive evolution of the virus is centered around its attachment and fusion protein(s) to maintain critical functions, and at the same time adapt the virus to diverse selective pressures, such as population‐level immunity and therapeutic interventions.

The SARS‐CoV‐2 spike (S) protein is a highly flexible and dynamic viral class I fusion surface glycoprotein, mediating both attachment to the host cell and virus‐cell fusion and therefore is the primary determinant of virus host‐range [3, 4]. On virions, spike is mainly present as a prefusion homotrimer [5], in which the hinge‐like regions within its S1 subunits enable motional flexibility of each of the individual receptor‐binding domains (RBDs) at the apex of the trimer. These can transition between “up” and “down” states; the “RBD‐up”‐state supporting ACE2 receptor engagement, while the RBD‐“down” configuration represents a receptor‐inaccessible state for most variants [6, 7].

We and others have shown that spike is capable of three‐valent attachment to up to three individual ACE2 receptors [6, 8]; the avidity and/or stability of this interaction being key strategies of the virus to modulate viral attachment to host cells [8]. Single mutations and particularly, the ensemble of mutations within the spike may not only alter local spike structures, but also long‐range modulations of the S dynamics and, hence, its conformational plasticity [9], with effects on its functional traits. Since the description of the first viral isolates by the end of December 2019, the SARS‐CoV‐2 virus has evolved substantially, diversifying into variants with distinct phenotypic attributes related to replication, transmission, virulence and immune evasion [10]. D614G was among the first significant mutations that emerged early in the pandemic [11, 12]. It enhanced transmissibility of the virus by increasing the proportion of RBDs in the ACE2‐accessible “up” state as well as S1/S2 proteolysis [13]. As infection and vaccination shaped the immune landscape of the population, novel, more heavily mutated variants have emerged. The WHO has assigned simple, easy‐to‐remember Greek labels for key variants that displayed genetic signatures posing a significant threat to the public or that were experimentally proven to be associated with increased transmissibility, enhanced disease severity, or reduced effectiveness of public health measures [14]. Early variants of concern (VOCs), such as Alpha (Pango lineage B.1.1.7), Beta (Pango lineage B1.351) and Gamma (Pango lineage P.1), were regionally dominant in Europe, southern Africa and South America, respectively, while Delta (Pango lineage B1.617.2) was the first VOC to become dominant worldwide, as reviewed by Carabelli et al. [15]. The emergence of Omicron marked a major epidemiological shift. Unlike preceding VOCs, Omicron gave rise to remarkable diversity, having evolved into several sublineages (incl BA1, BA2, BA3, BA4, BA5, XBB1, and others), with significant antigenic distance to pre‐Omicron strains and an altered entry pathway [16].

As the virus continues to evolve, a comprehensive understanding of the interplay between adaptive evolution of the spike protein and its conformational and motional plasticity reveals valuable insights into the mechanisms of viral evolution as well as opportunities for intervention. To gain a precise picture of the overall structural evolution of the SARS‐CoV‐2 spike protein with respect to its conformational mobility and motional dynamics in real‐time, we video‐recorded ten different spike proteins with high‐speed atomic force microscopy (HS‐AFM): Wuhan‐1 (W), Alpha (α), Beta (ß), Gamma (γ), Delta (δ) and Omicron BA1 (ο BA1), BA2 (ο BA2), BA5 (ο BA5), XBB1.5 (ο XBB1.5), and JN.1 (ο JN.1). Given that the spike proteins’ dynamic conformational landscape may modulate binding with its recognized receptor, we conducted single‐molecule force spectroscopy (SMFS) experiments with the ACE2 receptor expressed on Vero E6 cells. This grants a complementary approach that allows for direct quantification of unbinding forces and interaction kinetics between individual molecules and opens a unique window to reveal the mechanisms of the interaction between the spike and the viral entry receptor, which remain obscured in conventional bulk‐phase assays, such as surface plasmon spectroscopy and bilayer interferometry.

We found a pattern of differences in protein compactness and plasticity alongside the spike protein's evolutionary trajectory, suggesting that the protein evolved within an interplay of increasing host cell receptor binding affinity and evading the ever‐increasing immunity of the host. These patterns directly affected not only the valency of interaction with the ACE2 receptor, but also the stability of its interaction. Investigating such changes in viral surface proteins responsible for viral attachment to host cell receptors permits a detailed understanding of the virus’ structural evolution and mechanisms to evade immunity in later evolutionary stages, aiding rational Coronavirus vaccine development.

## 2 Results and Discussion

### 2.1 Recombinant Expression of the Ancestral SARS‐CoV‐2 Spike Protein and of Dominant Variants of Concern

**FIGURE 1:**
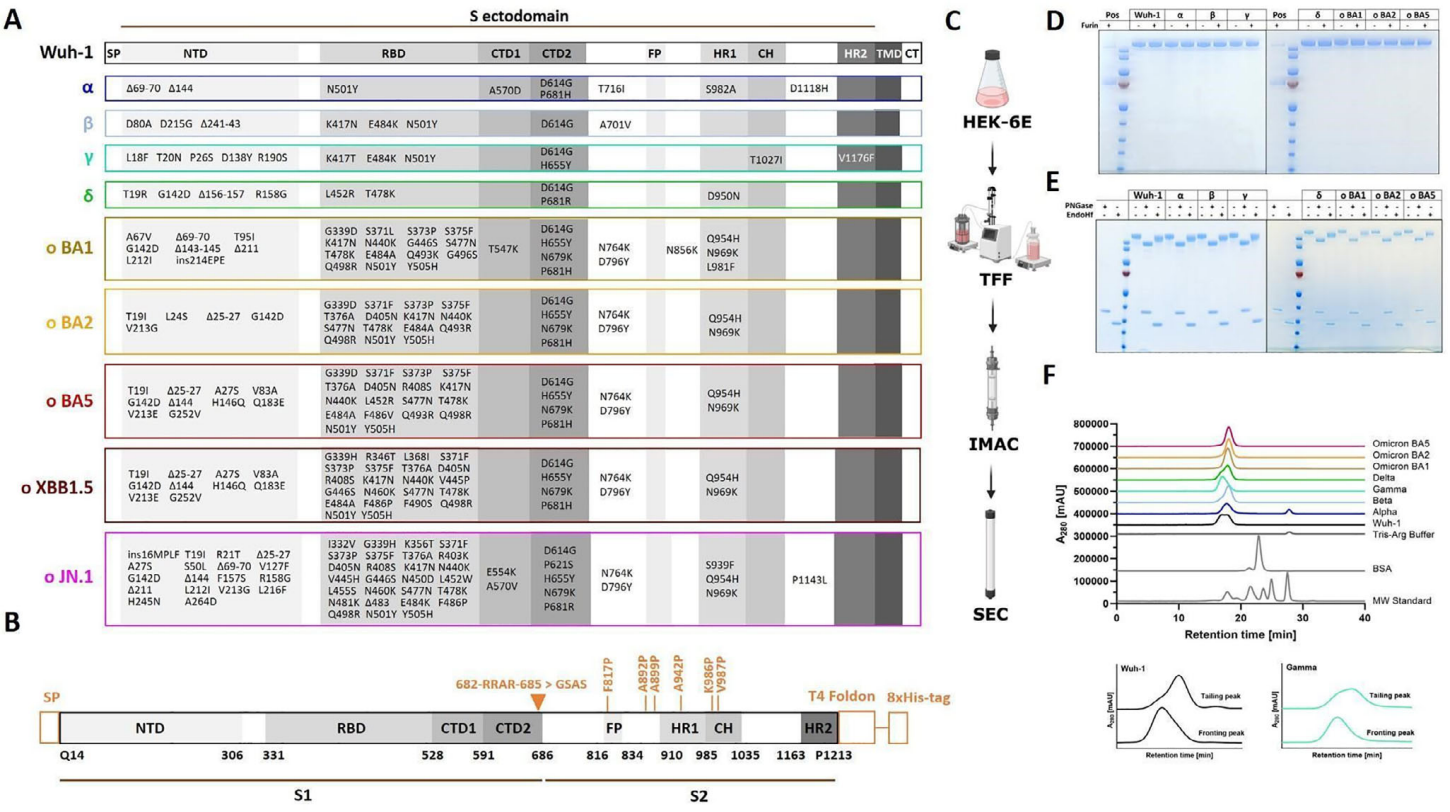
Recombinant expression and characterization of trimeric SARS‐CoV‐2 spike ectodomains. (A) Structural organization and mutations of spike variants relative to the ancestral Wuhan‐1 strain. The spike includes a signal peptide (SP), ectodomain, transmembrane domain (TMD), and cytoplasmic tail (CT). The ectodomain is cleaved into two structural subunits, S1 and S2. S1 contains the N‐terminal domain (NTD), receptor‐binding domain (RBD), C‐terminal domains 1 and 2 (CTD1 and CTD2), while S2 covers the fusion peptide (FP), heptad repeat 1 (HR1), central helix (CH), and heptad repeat 2 (HR2). (B) Schematic of the spike HexaPro ectodomain [17] used for protein design, incorporating six stabilizing proline mutations and an inactivated furin cleavage site. (C) Schematic of the spike production process, including TFF, tangential flow filtration, IMAC, immobilized metal anion chromatography, SEC, size exclusion chromatography. Omicron XBB.1.5 and JN.1 spikes were obtained commercially; see Methods. (D) SDS‐PAGE analysis of SEC‐purified spike proteins with (+) or without (‐) furin treatment confirms resistance to proteolytic cleavage. (E) Glycosylation analysis by SDS‐PAGE following PNGaseF or Endo*Hf* treatment to remove total or high‐mannose/hybrid glycans, respectively. (F, upper panel). HPLC‐SEC profiles of SEC‐purified S proteins on a Superose 6 Increase 10/300 GL with a BSA calibration standard and a molecular weight (MW) standard (from left to right: 670, 158, 44, 17, and 1.3 kDa (F, lower panel). Double peaks were resolved during preparative SEC, particularly for the Wuhan‐1 and γ spike, and were fractionated into a fronting and tailing part and analysed by HPLC‐SEC.

We investigated the evolutionary trajectory of the SARS‐CoV‐2 spike protein in terms of its conformational plasticity in prefusion state, starting from the spike of the first human isolate from December 2019 (Wuhan‐1) and covering the most dominant historic VOCs, including α, ß, γ, δ and early sublineages of the Omicron complex (ο BA1, ο BA2, ο BA5, ο XBB1.5, and ο JN.1). All variants have undergone significant adaptive evolution with an accumulative number of non‐synonymous mutations of about ten within the spike of the α to δ variant and between 30 and 64 mutations in the investigated ο sublineages (Figure 1A).

Recombinantly‐expressed, trimeric spike protein ectodomains have facilitated research on S structural features and its motional dynamics in solution, circumventing the need for working with infectious virus in high‐containment biological laboratories and detergent extraction of full‐length spike embedded in the lipid bilayer. The backbone design of the recombinant spike proteins generated in this study was based on the spike HexaPro ectodomain [17] (Figure 1B). HexaPro spikes cover six prefusion‐state‐stabilizing proline mutations within the S2 domain at amino acid positions 817, 892, 899, 942, and 986–987 (Wuhan‐1 numbering) and an abrogated furin cleavage site (_682_RRAR_685_> _682_GSAS_685_) [17] to prevent proteolytic cleavage at S1/S2 (Figure 1D), S1 shedding, and transition to postfusion conformation. The spike ectodomains were further equipped with a C‐terminal T4 foldon trimerization domain followed by an 8x‐histidine purification tag [2, 8]. Trimeric spike protein ectodomains were expressed in human embryonic kidney cells (HEK‐6E) and purified using a sequence of tangential‐flow filtration, immobilized metal anion exchange chromatography (IMAC), and size exclusion chromatography (SEC) (Figure 1C), yielding proteins of the highest purity as verified by SDS‐PAGE and high‐performance liquid chromatography (HPLC)‐SEC (Figure 1D,F). All proteins were extensively glycosylated, also including Endo*Hf*‐sensitive high‐mannose and/or hybrid glycan modifications (Figure 1E), as previously described [18].

On size‐exclusion chromatography, the Wuhan‐1, γ, and δ spike resolved as a double peak, or a peak with a pronounced shoulder, respectively (Figure 1F); an observation that has already been described by others [19]. We pooled the main fractions of the fronting and tailing parts of the peaks during preparative gel filtration for further analysis. We obtained too little yield of the purified and concentrated fractions of the δ spike peak shoulder; therefore, the whole, unfractionated peak was used for further analysis. Both entities of the Wuhan‐1 and γ double peak corresponded to the molecular weight of the spike trimer (Figure S1), as evaluated by size‐exclusion chromatography combined with multi‐angle light scattering (SEC‐MALS). Moreover, the fractions had a similar glycan profile (Table S1), as assessed by Liquid Chromatography‐Electrospray Ionization‐Mass Spectrometry (LS‐ESI MS). Collectively, these results suggested substantial differences in the overall conformation of the two spike species, affecting their hydrodynamic radii and hence, their retention during gel filtration. Individual RBDs within the S1 subunits of the spike homotrimer can reversibly transition between a receptor‐accessible up‐state, or an inaccessible down‐state. (Hydrogen‐Deuterium Exchange Mass Spectrometry) HDX‐MS data on the 2P‐S demonstrated that RBD transitions have no or little impact on the overall conformation of the membrane‐proximal S2 subunit of the uncleaved, 2‐proline‐(2P)‐stabilized prefusion S in the absence of receptor [20]. Therefore, we do not assume that RBD up‐down movements of the uncleaved variants within the HexaPro backbone used in this study account for the observed shift in the gel filtration retention profile. Next to the motional flexibility of individual RBDs, the overall structural organization of the prefusion spike is dynamic and interconverts between the canonical, compact prefusion structure resolved by cryo‐EM [21] and a more extended prefusion conformation, exposing the trimer interface [22]. Notably, these two spike prefusion conformations have already been resolved by Stuible et al. during analytical size‐exclusion chromatography and were investigated by HDX‐MS) [19]. There, the faster‐eluting entity of the 2P‐ spike corresponded to the open spike conformation, and the slower‐eluting entity corresponded to the more tightly packed prefusion state. Different spike variants were shown to occupy these conformations at variable magnitudes, with *wildtype* and γ predominantly presenting in open conformation, ß and δ predominantly in compact conformation, and α displaying a mixed phenotype. In a direct comparison of the 2P‐spike with the HexaPro design, Costello et al. [22] have demonstrated that HexaPro can adopt both conformations, but introduces a bias toward the canonical, more compact trimer, in alignment with improved stabilization of the prefusion conformation. To gain a better understanding of the two spike entities in relation to their conformational plasticity, as well as kinetics and valency of binding to the ACE2 receptor, we included them in our subsequent analyses.

### 2.2 Viral Evolution Affected Spike Protein Conformational Compactness and Structural Dynamics

We first set out to investigate how the ensemble of mutations that have evolved within the SARS‐CoV‐2 spike variants affected the motional dynamics and conformational compactness of the proteins. Using high‐speed atomic force microscopy [8, 23, 24, 25] as a method to observe dynamic changes of proteins with sub‐nm spatial and 50‐ms temporal resolution in an aqueous environment, we video‐recorded the proteins that had been gently adsorbed onto mica surfaces to maintain their conformational mobility in the prefusion state.

**FIGURE 2:**
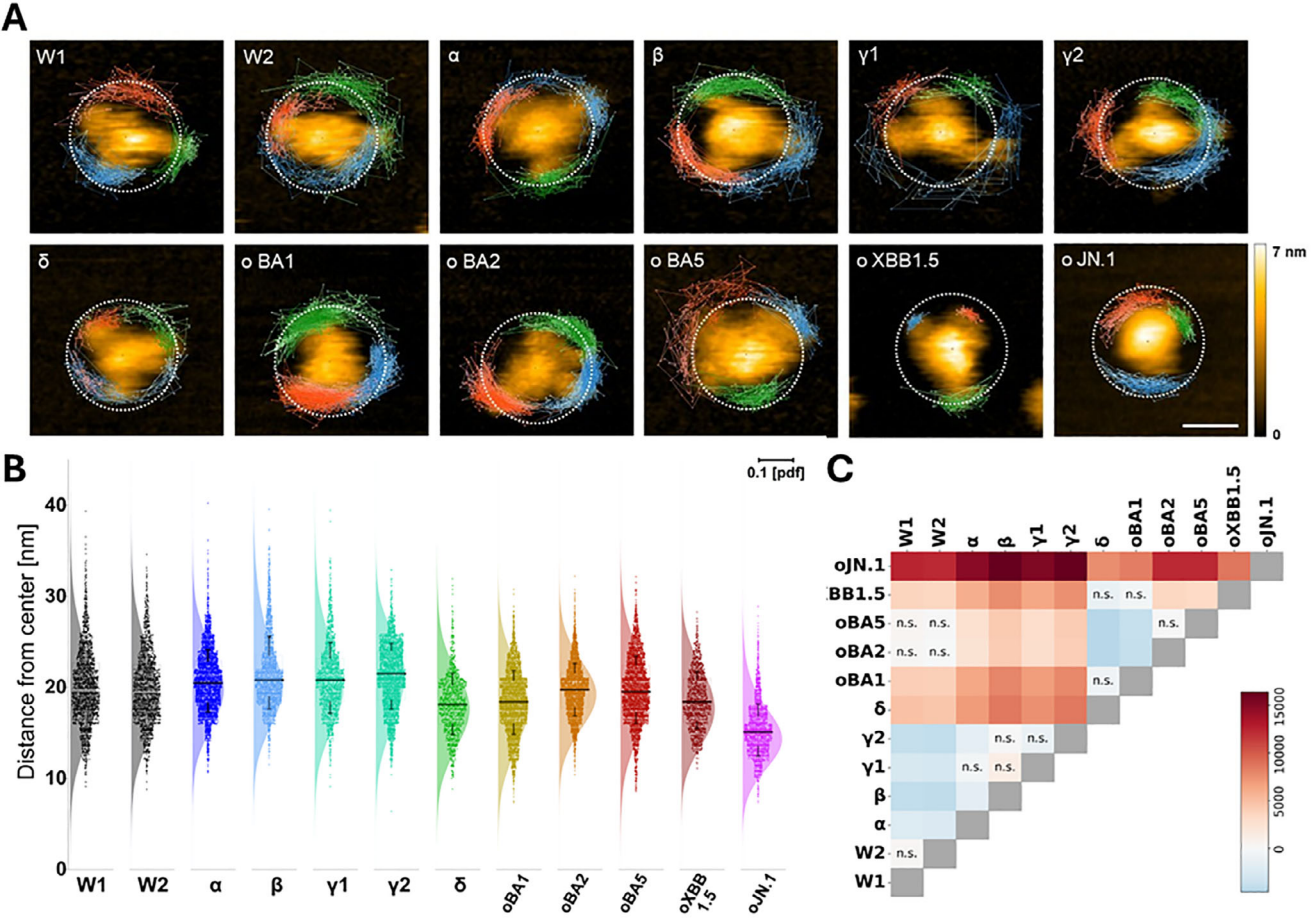
The spike proteins of the investigated variants show differences in lateral RBD dynamics and extension. (A) Representation for the cumulative movements of the three individual RBDs (RBD apexes tracked using red, green and blue dots, respectively) of the investigated spike variants (scalebar 20 nm; dashed circle r = 20 nm for visual aid). (B) RBD distances from the center of mass are portrayed as boxplots, with individual datapoints displayed in scatterplots and overlaid with their probability density functions. The distributions for the RBD extensions were derived from video‐recordings of thermal movements of the spike, and the large standard deviations are therefore representative of the stochasticity of the process. (C) Development of RBD extensions throughout viral evolution. This presentation displays the tendencies for larger or smaller RBD extensions relative to groupwise comparisons of the ten variants. Statistical testing was done using the Kruskal‐Wallis ANOVA and the post hoc Dunn's test for pairwise comparison. Mean rank differences gained from pairwise comparisons of the post hoc Dunn's test [41] were plotted in a 2‐D false color map, whereby negative (blue‐shaded) mean rank differences indicate that spike variants displayed on the *x*‐axis have a significantly smaller RBD extension compared to the variants on the *y*‐axis, while red colors indicate positive mean rank differences and a larger RBD extensions on variants on the *x*‐axis compared to the *y*‐axis. The false color scale bar represents the mean rank differences. (N(Wuhan‐1 peak 1) = 2319, N(Wuhan‐1 peak 2) = 1863, N(ɑ) = 3855, N(β) = 2697, N(γ peak 1) = 2127, N(γ peak 2) = 4287, N(δ) = 1335, N(o BA1) = 5190, N(o BA2) = 4839, N(o BA5) = 3516, N(o XBB1.5) = 1482), N(o JN.1) = 3117). n.s indicates that the compared datasets do not significantly differ (ɑ = 0.001).

Individual RBDs of the homotrimer of all spike variants considerably extended from the spike center of mass and showed fast arc‐like movements (Figure 2A, see also Movies S1–S12 **for the raw data**). The HS‐AFM video recordings served to depict the average lateral RBD displacement speeds of the expressed spike variants. We found the RBD displacement speed to be independent of the number of stabilizing proline mutations, as the Wuhan‐1 spike within the HexaPro backbone displayed comparable values as the 2‐proline spikes previously reported by us [8]. Over the SARS‐CoV‐2 evolution, we observed a gradual reduction in the lateral speed from 48.12 nm/s for Wuhan‐1 peak 1 to 30.54 nm/s for o BA1, followed by a slight increase for o BA2 and o BA5 with RBD speeds of 36.60 nm/s and 37.76 nm/s, respectively. Omicron XBB1.5 and JN.1 marked an exception with an apparent impaired RBD flexibility. This was reflected by the slowest RBD movements overall (o XBB1.5: 23.52 nm/s; o JN.1: 24.25 nm/s), which were less than half of the lateral speed of both Wuhan‐1 spike proteins fractionated during SEC analysis (*p* < 0.001, Figure S2A,B and Tables S2 and S3). The simultaneously evaluated average rotational displacement of the RBD arms in between frames (160 ms/frame) of the Wuhan‐1 spike protein was about 20 degrees in our measurements (Table S4), and the variations in rotational speeds among the spike variants followed a similar, but less pronounced trend as the lateral speeds (Figure S2C,D and Table S5).

We then tracked the extensions of the RBD apexes from the centers of mass of individual video‐recorded spike proteins (Figure 2A), which were representative of the level of protein compactness. RBD extensions ranged from 10 to 40 nm for the Wuhan‐1 spike protein, with no significant differences between the two SEC‐resolved spike entities (mean W1: 20.72 ± 3.42 nm vs. W2: 19.95 ± 3.96 nm, n.s., Figure 2B and Tables S6 and S7). Interestingly, the mean RBD distance from the center of mass in the RBD‐up conformations retrieved from several Wuhan‐1 cryo‐EM structures was considerably smaller (4.8 ± 2 nm, *n* = 23) [8, 20, 26, 27, 28, 29] than we observed with HS‐AFM in near‐physiological buffer. This suggests that the RBDs can extend more widely from the spike protein center of mass when in contact with a surface.

RBD extensions from the center of mass changed significantly throughout the spike evolution (Figure 2B and Tables S6 and S7), which was visualized through pairwise comparisons between the spike variants via a heatmap (Figure 2C). We observed small (< 1 nm), but gradual increases in RBD extensions along the evolutionary trajectory up to the γ variant (*p* < 0.001, Figure S2A,B and Table S6), suggesting a gradual opening of the spike protein in the early viral evolution. Increasing levels in RBD‐up conformations in variants following Wuhan‐1, possibly facilitated by the D614 mutation [13, 30, 31], have been previously reported by others and support our findings. Of note, the appearances of δ and the Omicron sublineage BA1 reverted this trend and were accompanied by restrictions in RBD extensions (δ: 18.22 ± 3.35 nm, ο BA1: 18.35 ± 3.49 nm), when compared to the preceding α, ß and γ variants (*p* < 0.001, Table S7). Omicron sublineage spikes generally have been described to adopt more compact conformations by forming new interdomain and inter‐subunit interactions [8, 30, 32, 33] and to predominantly adopt 1‐up RBD states [13, 34, 35, 36]. In particular, mutations found within spike o BA1 were shown to be associated with a much tighter packing of the RBDs and the S2 central helices [37], which is in good agreement with our findings. Remarkably, RBD expansions in the subsequent o BA2 and o BA5 variants again increased relative to o BA1 (*p* < 0.001) and aligned back to Wuhan‐1 levels (for both W1 and W2 compared to o BA2 and o BA5, *p* > 0.001), Figure 2C ‘n.s.’). This reversion to conformational relaxation could have been supported by the relaxation of the S2 helices, which evolved to increase protein stability, as reviewed by Parsons & Acharya [37]. RBD extensions decreased again in o XBB1.5 and, finally, o JN.1 was found to be the most compact among all investigated spike variants, featuring the lowest range of RBD extensions from the protein center of mass, with 15.33 ± 2.87 nm (*p* < 0.001, Table S7). Of note, cryo‐EM data and MD simulations of the JN.1 spike protein reveal prefusion conformations with features suggestive of enhanced interprotomer stabilization and compactness [38, 39, 40], indicative of a less dynamic protein phenotype, such as we have observed. The proteins’ RBD extensions from the center of mass correlated well with the proteins’ inter‐RBD distances (Pearson's R = 0.88, p = 0.0002; Figure S3), which further underpins the evolution of protein compactness derived from our HS‐AFM data.

### 2.3 Spike Variants Differ in Their Capacity for Multivalent ACE2‐Binding

**FIGURE 3:**
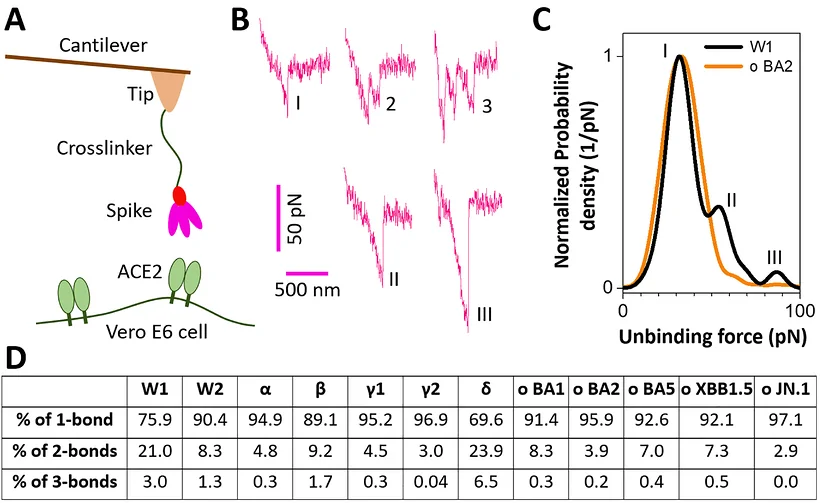
The spike proteins of the investigated VOCs differ in their capacity for multivalent ACE2 binding. (A) The spike is conjugated onto the AFM cantilever tip through a flexible PEG cross‐linker for the force measurements with the ACE2 receptor expressed on Vero E6 cells. (B) The spike can form one, two, or three bonds with the ACE2 receptor. Upon rupture, the bonds can be separated sequentially (curves 2 and 3) or simultaneously (curves I and III). Force curves in this panel are from the Wuhan‐1 spike peak 1 (faster‐eluting peak fraction in size‐exclusion chromatography). (C) The probability density of the unbinding force from Wuhan‐1 peak 1 (W1, black curve) shows three peaks corresponding to the rupture of single, double, or triple bonds, with unbinding forces of 31.7 ± 7.7 pN, 53.9 ± 7.4 pN, and 86.7 ± 4.4 pN, respectively. In comparison, the probability density of the unbinding force from Omicron BA2 (o BA2, orange curve) shows much lower probabilities for double or triple bonds. The major peak corresponds to a single bond, with an unbinding force of 32.7 ± 10.4 pN. (D) Summary of the percentages of single, double, or triple bonds formed between the respective spike proteins and the ACE2 receptor on Vero E6 cells.

Having established that the spike variants differ in their conformational compactness and motional plasticity, we aimed to characterize how these traits might translate into the multivalent binding kinetics and affinity for the ACE2 receptor. Single‐molecule force spectroscopy conducted using AFM allows for the investigation of interactions at the single‐molecule level for direct quantification of unbinding forces and interaction kinetics between individual molecules. Spike ectodomains were conjugated onto AFM cantilever tips and brought into contact with ACE2 endogenously expressed on the surface of Vero E6 cells (Figure 3A). Subsequent retraction of the cantilever resulted in single bond breakages and enabled detection of specific unbinding forces with a few‐piconewton sensitivity. We found that the Wuh‐1 spike is capable of three‐valent binding to ACE2 on Vero E6 cells (Figure 3B). The interaction was ruptured sequentially (curves 2 and 3 in Figure 3B) or simultaneously (curves II and III in Figure 3B), and the statistical distribution of the unbinding force showed three local maxima for Wuhan‐1 peak 1 (W1, Figure 3C, black curve), corresponding to the rupture of single, double, or triple spike‐ACE2 bonds, respectively. The spike variants significantly differed in their ability to establish multiple bonds with the ACE2 receptor, with the δ variant being most potent in multivalent binding, establishing 23.9% double bonds and 6.5% triple bonds with the receptor (Figure 3D). A higher probability of forming double bonds also qualified the protein for 3‐bond formation with ACE2 (Figure S7). On the contrary, the α, γ, and Omicron sublineage spikes (e.g., o BA2, Figure 3C, orange curve) were among the proteins with low probabilities for multivalent ACE2 binding (cumulative all less than 8.6%, Figure 3D). Of which, o JN.1 was least capable of forming double bonds with the ACE2 receptor (2.9%) among all variants and did not form any triple bonds at all (Figure 3D). Interestingly, comparing the valency of interactions of Wuhan‐1 between the faster‐ and the slower‐eluting SEC fractions, we found that the faster‐eluting fraction of Wuhan‐1 was more capable of establishing multiple bonds with the ACE2 receptor (W1 vs. W2: 21% double bonds and 3% triple bonds vs. 8.3% double bonds and 1.3% triple bonds, Figure 3D).

Collectively, our data show that the spike proteins of the investigated VOCs differ in their capacity to establish multiple bonds with the ACE2 receptor expressed on Vero E6 cells, with the δ variant spike being particularly qualified for multivalent interaction and o JN.1 being the phenotype most shifted toward a single‐interaction phenotype.

### 2.4 The Late VOCs Improved Their ACE2 Binding Affinity Primarily via Prolonging the Interaction Stability

**FIGURE 4:**
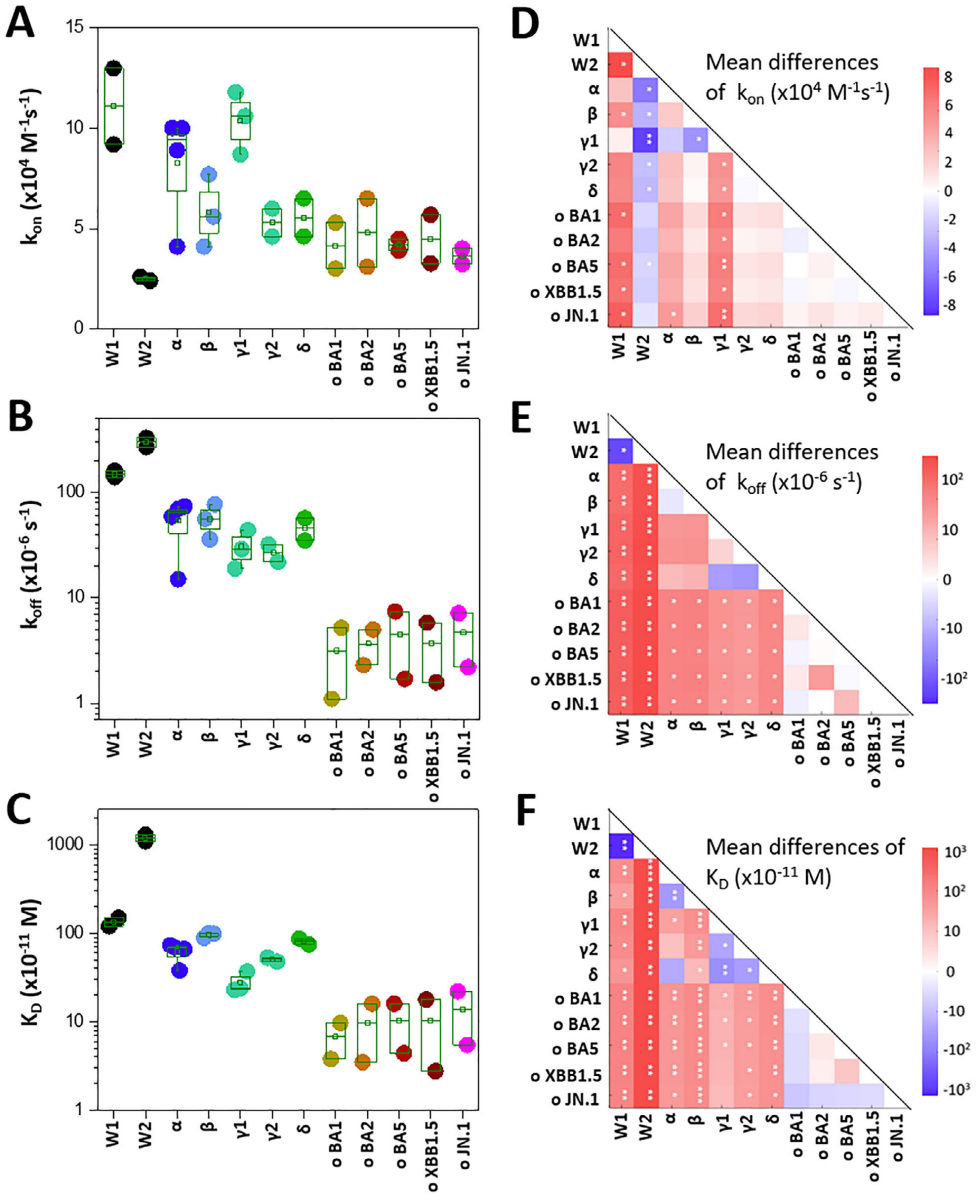
Variant‐specific differences in the binding affinity toward the ACE2 receptor are mainly due to different k_off_ rates. (A–C) The k_on_, k_off_, and K_D_ values extracted from the force measurements between the spike variants and ACE2. Each data point in a‐c corresponds to the measurement result from one AFM tip. The numbers of AFM tips and force curves for each variant are as follows: W1: two tips, 3800 curves; W2: two tips, 6000 curves; α: four tips, 13 200 curves; ß: three tips, 5400 curves; γ1: three tips, 6800 curves; γ2: two tips, 9600 curves; δ: two tips, 6600 curves; ο BA1: two tips, 4800 curves; ο BA2: two tips, 4100 curves; ο BA5: two tips, 4800 curves; ο XBB1.5: two tips, 6000 curves; ο JN.1: two tips, 7600 curves. The raw data of binding activity vs. contact time and unbinding force vs. force loading rate for calculating the k_on_ and k_off_ values are shown in Figures S4 and S6, respectively. (D–F) Mean differences of k_on_, k_off_, and K_D_ among the variants. The red color indicates that the variant from the *x*‐axis has higher k_on_, k_off_, or K_D_ than the variant from the *y*‐axis. The blue color indicates that the variant from the *x*‐axis has lower k_on_, k_off_, or K_D_ than the variant from the *y*‐axis. The white color indicates no difference. P‐values are from one‐tailed t‐test. One * indicates *p* < 0.05; two ^*^: *p* < 0.01; three ^*^: *p* < 0.001; four ^*^: *p* < 0.0001.

We finally set out to characterize the kinetics of interaction between the spike protein variants and the ACE2 receptor on Vero E6 cells (Figure 4 and Table 1). A key strength of SMFS lies in the precise experimental control it offers. Parameters including pulling velocity and contact time can be systematically varied. This level of control enables the extraction of kinetic association and dissociation rate constants of spike‐ACE2 interactions at the single‐molecule level. First, kinetic on‐rates (k_on_) were extracted (Figure 4A,D) from the increase in binding activity with cell surface contact time (Figure S4), by approximating the relation with pseudo‐first‐order kinetics. We observed subtle differences in the kinetic on‐rates between the variants, which were generally in the range of 3.6 × 10^4 ± 0.2^ m ^−1^s^−1^ (o JN.1) to 8.3 × 10^4 ± 0.2^ m ^−1^s^−1^ (α), and with a tendency for a decrease in the k_on_‐rates along the evolutionary trajectory. The fronting (W1 and γ1) and tailing (W2 and γ2) fractions collected from the double‐peak that had resolved during SEC‐purification of the Wuhan‐1 and γ variant were individually investigated. Interestingly, both Wuhan‐1 and γ spikes in peak 1 had the highest kinetic on‐rates among all tested variants, and k_on_‐rates were also significantly higher than those from the respective peaks 2 (W1: 1.1 × 10^5 ± 0.1^ m ^−1^s^−1 ^vs. W2: 2.5 × 10^4 ± 0.1^ m ^−1^s^−1^ and γ1: 1.0 × 10^5^ ^± 0.1^ m ^−1^s^−1 ^vs. γ2: 5.3 × 10^4 ± 0.1^ m ^−1^s^−1^; Figure 4A,D and Table 1), which was also observed for their kinetics of double bond formation (W1: 63 s^−1^ vs. W2: 11 s^−1^ and γ1: 23 s^−1 ^vs. γ2: 15 s^−1^; Table 1 and Figure S5). Thus, the Wuhan‐1 and γ spikes collected as faster‐eluting fractions of the double‐peak bound ACE2 more rapidly than those from the slower‐eluting fractions. Combined with our findings that the faster‐eluting fractions also bound ACE2 with higher valency (Figure 3D), these observations strengthen our hypothesis that the faster‐eluting spike proteins have a more stretched‐out conformation, facilitating quicker ACE2 binding. A high percentage of multiple bonds and a high kinetic on‐rate for double bond formation (65 s^−1^) were also found for the δ variant. Apparently, the ability for quicker binding better supports the formation of multiple bonds. Both, the specific amino acid residues at the binding site and the variation of the distribution of the glycosylation might contribute to this effect.

**TABLE 1:** Summary of the kinetic rates of the interaction between the different spike protein variants and the ACE2 receptor on Vero E6 cells.

	k_on_ (M^−1^s^−1^)	k_off_ (s^−1^)	K_D_ (M)	k_2_ (s^−1^)	k_3_ (s^−1^)
**W1**	1.1 × 10^5 ± 0.1^	1.5 × 10^−4 ± 0.1^	1.4 × 10^−9 ± 0.1^	6.3 × 10^1 ± 0.2^	2.1 × 10^1 ± 0.3^
**W2**	2.5 × 10^4 ± 0.1^	3.0 × 10^−4 ± 0.1^	1.2 × 10^−8 ± 0.1^	1.1 × 10^1 ± 0.4^	—
**α**	8.3 × 10^4 ± 0.2^	5.4 × 10^−5 ± 0.2^	6.2 × 10^−10 ± 0.2^	3.2 × 10^1 ± 0.2^	—
**β**	5.8 × 10^4 ± 0.2^	5.6 × 10^−5 ± 0.2^	9.6 × 10^−10 ± 0.1^	4.9 × 10^1 ± 0.2^	—
**γ1**	1.0 × 10^5 ± 0.1^	3.1 × 10^−5 ± 0.2^	2.8 × 10^−10 ± 0.2^	2.3 × 10^1 ± 0.4^	—
**γ2**	5.3 × 10^4 ± 0.1^	2.7 × 10^−5 ± 0.2^	5.1 × 10^−10 ± 0.1^	1.5 × 10^1 ± 0.1^	—
**δ**	5.6 × 10^4 ± 0.1^	4.6 × 10^−5 ± 0.2^	8.1 × 10^−10 ± 0.1^	6.5 × 10^1 ± 0.1^	1.8 × 10^1 ± 0.4^
**ο BA1**	4.2 × 10^4 ± 0.2^	3.2 × 10^−6 ± 0.3^	6.8 × 10^−11 ± 0.3^	3.9 × 10^1 ± 0.3^	—
**ο BA2**	4.8 × 10^4 ± 0.2^	3.7 × 10^−6 ± 0.2^	9.8 × 10^−11 ± 0.3^	3.7 × 10^1 ± 0.4^	—
**ο BA5**	4.2 × 10^4 ± 0.1^	4.6 × 10^−6 ± 0.3^	1.0 × 10^−10 ± 0.3^	2.6 × 10^1 ± 0.3^	—
**ο XBB1.5**	4.5 × 10^4 ± 0.2^	3.7 × 10^−6 ± 0.3^	1.0 × 10^−10 ± 0.4^	2.1 × 10^1 ± 0.3^	—
**ο JN.1**	3.6 × 10^4 ± 0.1^	4.7 × 10^−6 ± 0.3^	1.4 × 10^−10 ± 0.3^	1.3 × 10^1 ± 0.2^	—

For attaining the kinetic dissociation rates, we first obtained the relation between the unbinding forces and the force loading rates for tips functionalized with the different prefusion‐stabilized spike ectodomains from force measurements performed with a series of different pulling velocities (Figure S6). By computing these data sets using the Bell‐Evans model, we then obtained the kinetic off‐rate (k_off_) (Figure 4B,E). The early variants (α, ß, γ, and δ) displayed similar k_off_ values in the range of 2.7 × 10^−5 ± 0.2^ s^−1^ to 5.6 × 10^−5 ± 0.2^ s^−1^, which were all significantly lower than the average k_off_ of Wuhan‐1 (2.3 × 10^−4 ± 0.2^ s^−1^, p = 1E‐6, one‐tailed t‐test). This suggests that the bonds established between the spike of the early VOCs support an up to 4‐fold longer stability with the ACE2 receptor than established by the ancestral Wuhan‐1 spike. The Omicron sublineages display even lower kinetic off‐rates than the preceding variants, with an average k_off_ value of 3.9 × 10^−6 ± 0.3^ s^−1^. This translates into an average bond stability increase of about 50 times between Omicron spikes and ACE2 compared to that of Wuhan‐1. Differences in kinetic off‐rates between the SEC‐resolved peaks of Wuhan‐1 and γ were less pronounced than for the kinetic on‐rates, which were 1.5 × 10^−4 ± 0.1^ s^−1^ and 3.0 × 10^−4 ± 0.1^ s^−1^ for W1 and W2 and 3.1 × 10^−5 ± 0.2^ s^−1^ and 2.7 × 10^−5 ± 0.2^ s^−1^ for γ1 and γ2, respectively (Figure 4 and Table 1). Combining the k_off_ and k_on_ values, we retrieved the dissociation constant K_D_ for the Spike:ACE2 interaction (Figure 4C,F), which decreases along the evolutionary trajectory. For α, ß, γ, and δ, the average K_D_ value, 6.3 × 10^−10 ± 0.2^ m is significantly lower than that of Wuhan‐1, 6.7 × 10^−9 ± 0.3^ m (p = 1E‐3, one tailed t‐test) and significantly higher than that for the Omicron spikes, 1.0 × 10^−10 ± 0.3^ m (p = 1E‐5, one tailed t‐test).

Collectively, these kinetic data suggest that the gradual chronologic increase in ACE2 binding affinity is largely mediated by increasing the interaction stability between the spike and ACE2 (Figure 4, Table 1 and Figure S7).

## 3 Cumulative Conclusions

While the majority of studies have primarily focused on the interaction of the SARS‐CoV‐2 spike RBDs with the viral entry receptor ACE2, there remains limited understanding of how mutations intrinsically influence the motional dynamics of the whole spike homotrimer and how these shape the context of receptor binding. High‐speed atomic force microscopy and single‐molecule force spectroscopy enabled real‐time, nanoscale interrogation of protein conformational dynamics under near‐physiological conditions, offering superior spatiotemporal resolution, and revealed transient intermediates, force‐dependent transitions, and heterogeneous association and dissociation pathways that remain largely inaccessible to ensemble‐averaged or static structural techniques.

Our high‐speed AFM data indicate a conformational compactness change of the spike protein in its prefusion form in a non‐monotonic, lineage‐specific manner, with a trend for conformational opening in early variants of concern (up to γ) to slower RBD motion and increased conformational compactness in the later VOCs, starting with δ. Across the evolutionary timeline of the Omicron complex, we observed a dynamic structural tuning of the protein's conformational and motional plasticity (Figure 2), likely to balance ACE2 engagement, immune evasion, and structural stability. In agreement with previous results, we observed that the early Omicron sublineage BA.1 adopted a highly compact and tightly packed conformation by forming new interdomain and inter‐subunit interactions [30, 32, 33, 37, 42], followed by a partial relaxation in subsequent Omicron sublineages (o BA.2, o BA.5) and reversion to compact, less mobile conformations for the o XBB1.5 and o JN.1 lineages. In fact, a trend for enhanced inter‐protomer stability and protein compaction in o JN.1 has also been revealed by another cryo‐EM study [39] and by (all‐atom) MD simulations [38, 40], which identified o JN.1 to exhibit enhanced structural stability and compaction as compared to the Wuhan‐1 spike. This shift is likely supported by the formation of new contacts between mutated–mutated or mutated–conserved residues [38], as well as stabilizing changes in the S2 region, and suggests mechanical/dynamic properties as selectable traits that support the modulation of RBD mobility and packing to meet changing immune and transmission pressures.

More open plastic spikes are expected to have larger RBD lateral reaches, as well as a higher probability of multiple RBDs being simultaneously accessible, and thus, better support multivalent and faster ACE2 engagement (high kinetic on‐rates). We wished to assess whether our HS‐AFM data on spike protein plasticity can directly inform us how easily the protein samples receptor‐accessible states and sets opportunities for receptor binding. The Wuhan‐1 and γ spike conformations collected from the faster‐eluting fractions of the SEC double peak (Figure 1), which were previously identified by HDX mass spectrometry as the more open spike conformations [19], displayed higher levels of multivalent ACE2 interaction and faster kinetic on‐rates with ACE2 expressed on Vero E6 cells. In contrast, pronounced spike rigidification, as observed for Omicron JN.1, was associated with the lowest capacity for multivalent ACE2 engagement and the slowest k_on_ with ACE2 (Figure 3 and Table 1). Although our integrated HS‐AFM/SMFS observations across all ten spike variants largely followed this trend, the data do not support the conclusion that the extent of spike conformational plasticity alone strictly determines either k_on_ or the valency of interaction with the ACE2 receptor. Kinetic on‐rates and binding valency are likely also governed by the effective encounter time between ACE2 and binding‐competent RBD states and the intrinsic chemical affinity of the ACE2–RBD interface, which together determine productive receptor engagement.

Our SMFS results strengthen the assumption that SARS‐CoV‐2 variants appear to have explored different kinetic regimens to the same functional problem, ensuring sufficient ACE2‐binding strength and residence time for viral entry. Delta appears to favor multivalent receptor engagement, consistent with a model in which rapid rebinding and higher local avidity arise from simultaneous or sequential occupancy of multiple ACE2 molecules on the cell surface. In contrast, Omicron exhibits slower dissociation from ACE2 at the level of individual spike‐receptor contacts, implying a more stable binary complex despite reduced multivalent engagement. These key distinctions may be grounded in the distinct entry pathways, which integrate binding and dissociation kinetics with protease usage to optimize infectivity across diverse cellular contexts. Delta relies more heavily on TMPRSS2‐driven plasma membrane fusion, where its propensity for multivalent ACE2 capture facilitates rapid receptor clustering and avidity enhancement on the cell surface, promoting efficient cleavage and fusion under conditions of low or moderate ACE2 density [43, 44]. In contrast, Omicron variants shifted predominantly toward endocytic entry, exhibiting reduced TMPRSS2‐dependence and lower fusogenicity [44]; here, its slower ACE2 dissociation kinetics may stabilize individual spike‐receptor contacts during trafficking through the endosomal compartment, likely compensating for weaker surface‐level multivalency and enabling cathepsin‐mediated activation in a pathway less sensitive to TMPRSS2 availability.

Together, the complementary experimental approaches used in this study establish a comprehensive framework for investigating the molecular determinants of spike protein dynamics and ACE2 engagement. The following considerations place these observations in the context of the native virus–host interface and highlight opportunities for future investigation. Under our HS‐AFM imaging conditions, spike proteins adsorb to negatively charged mica in the presence of divalent cations (MgCl_2_, CaCl_2_ and NiCl_2_), raising the possibility that electrostatic interactions with the substrate could influence RBD conformational dynamics. This question was addressed in our previous work by imaging the N234Q glycomutant under identical experimental conditions [8]. The N234 glycan has been shown to stabilize the open RBD conformation, and substitution of this residue (N234Q) strongly shifts the conformational equilibrium toward the all‐RBD‐down state. Consistent with these reports, HS‐AFM exclusively detected the N234Q mutant in the closed conformation, indicating that electrostatic interactions between the spike protein and the mica surface are insufficient to induce RBD opening. Furthermore, although SARS‐CoV‐2 spike proteins have acquired a progressively more positive surface charge during viral evolution [10, 45, 46, 47, 48], we observed a reduction rather than an increase in RBD mobility across variants despite imaging on divalent ion‐treated mica. Together, these findings suggest that evolutionary changes in spike surface charge do not substantially influence the interaction with the substrate and are unlikely to account for the variant‐dependent differences in RBD dynamics observed in this study.

The controlled imaging conditions were complemented by the use of a standardized recombinant spike platform, which enables direct comparison across variants while introducing its own experimental context. Our measurements were performed using recombinant trimeric spike ectodomains stabilized in the prefusion state on a HexaPro/Δfurin background, in which the native transmembrane and cytoplasmic regions were replaced by a T4 fibritin trimerization domain. Although such constructs are widely used and enable robust structural and biophysical analyses, they may not fully recapitulate the conformational landscape, proteolytic processing, or membrane‐dependent effects of spike on virions or infected cells. Among the engineered features, the furin cleavage site is expected to have the most pronounced functional consequence on the conformational landscape of the spike protein [49, 50, 51]. The furin cleavage site is not merely a proteolytic processing element but a key regulator of spike function. Cleavage at the S1/S2 junction allosterically destabilizes the S2′ region, promotes sampling of the open trimer conformation and allosteric communication within the protein, promoting the RBD transitions [49, 51]. Consequently, cleavage‐site‐deficient spike constructs may not fully recapitulate the structural and functional properties of native spike and slightly underestimate interface opening and RBD motility relative to native, cleavage‐competent spike. The introduction of proline mutations within the spike S2 subunit increases spike rigidity and prefusion stability, thereby reducing conformational plasticity and reshaping allosteric communication within the trimer toward a more constrained, activation‐resistant state [17]. HDX‐MS analyses suggest that the two stabilizing proline residues do not introduce large‐scale changes to the overall structure and flexibility of the spike protein, but can affect steady‐state populations of the open/closed trimer [50] and the RBD opening propensity [52]. In particular, K986P has been reported to favor RBD opening by weakening S1–S2 subunit contacts [52]. The spike variant ectodomains analysed within this study are based on the HexaPro backbone [17], which introduces four additional prolines into the S2 subunit, increasing recombinant protein expression and thermal stability. Importantly, cryo‐EM studies have shown that HexaPro can still access open conformations, including a two‐RBD‐up state [17]. This suggests that the additional prolines do not simply lock the spike in a closed conformation, but rather stabilize the overall prefusion architecture, while retaining conformational flexibility of the RBD, permitting RBD opening [17]. Taken together, the combined effects of furin‐site abrogation and prefusion stabilization suppress S1/S2 cleavage and S1 shedding, thereby likely shifting activation thresholds and conformational equilibria. These modifications are likely not neutral with respect to the RBD dynamics, and we assume they may reshape the conformational landscape by allosteric mechanisms, including the population of receptor‐accessible open states we observed.

Replacement of the native transmembrane domain with a trimerization domain preserves trimeric assembly, but modestly alters trimer stability. In matched comparisons using sequence‐identical eVLP spike constructs, membrane‐anchored and soluble spike proteins displayed the same overall conformational repertoire and similar opening rates, although the membrane context modestly favored closed‐interface spike states [50]. Soluble trimers lack a lipid bilayer, lateral membrane confinement, and native spike density, all of which can influence ACE2 avidity and engagement geometry. We assume that membrane anchoring and the crowded membrane context likely quantitatively dampen the amplitude of RBD movements we see on mica, which we assume to represent an upper bound. In addition, secreted ectodomains may exhibit site‐specific glycan processing that differs from virion‐ or cell‐surface‐associated spike, potentially altering interaction dynamics.

Although our recombinant spike constructs may not fully capture the quantitative dynamics of native spike on virions, our conclusions are based on relative comparisons within a single, well‐controlled protein scaffold, thereby minimizing confounding effects of construct heterogeneity. We therefore interpret the observed motions as native‐like conformational features that are accessible on membranes, while acknowledging that state populations and movement amplitudes may slightly differ in the virion.

Whereas the recombinant constructs define the conformational landscape that can be sampled, the force spectroscopy experiments determine how these interactions are interrogated. In terms of interaction strength and kinetics with the ACE2 receptor on Vero E6 cells, we found that the engineered trimeric Wuhan‐1 spike ectodomain (Wuhan‐1 pooled) behaves comparable to the spike within the membrane context of the whole virus (inactivated Wuhan‐1 strain) (Figure S8 and Table S8).

The SARS‐CoV‐2 spike–ACE2 interaction is driven by a multivalent, cooperative mechanism that is suggested to shape clustering on both the viral and cellular sides. Our force spectroscopy assay was configured to resolve receptor engagement by individual spike trimers. The AFM tip functionalization strategy resulted in a cross‐linker density of fewer than 1000 molecules µm ^−^^2^ [53], corresponding to an average intermolecular spacing exceeding 30 nm. Given the ∼10 nm radius of the cantilever tip (Bruker MSCT), only spike proteins located near the tip apex are expected to access ACE2 receptors on the cell surface. In addition, the maximum applied force during force spectroscopy measurements was limited to approximately 30 pN, minimizing cell membrane indentation and reducing the likelihood that spike molecules positioned away from the apex could contact the cell. Under these conditions, simultaneous engagement of multiple spike trimers is not expected. Consistent with this interpretation, tetravalent binding events were not observed in the present study, indicating that the detected double‐ and triple‐bond rupture events most likely arise from multivalent ACE2 engagement with a single spike trimer. This conclusion is further supported by two control experiments. First, cantilevers functionalized with monomeric RBD yielded exclusively single‐bond rupture events, demonstrating that higher‐order interactions require the trimeric spike architecture [8]. Second, functionalization with intact SARS‐CoV‐2 virions markedly increased the frequency of double‐ and triple‐bond events and additionally yielded tetravalent to hexavalent interactions, as expected when multiple spike trimers are simultaneously available for receptor engagement [54]. Together, these observations support the interpretation that the multivalent interactions quantified here predominantly reflect receptor binding by individual spike trimers rather than cooperative binding by multiple spikes.

The molecular interactions resolved here occur within a broader membrane context that contributes to viral attachment and entry. Our SMFS measurements capture the behavior of individual spike trimers, whereas viral entry emerges from collective interactions between multiple receptors and spikes on opposing membranes. ACE2 receptors diffuse laterally through the lipid bilayer membrane and gather at the viral contact point [55, 56, 57]. Our system, based on VeroE6, shares the same cellular characteristics, as it was previously reported that lateral diffusion of the ACE2 receptor is influenced by its association with cholesterol‐rich lipid rafts and the cortical cytoskeleton [58]. Furthermore, the SARS‐CoV‐2 spike uses sialic acid moieties on the cell membrane as a binding anchor, which increases the residence time of the virus on the cell surface and aids in the binding of the main receptor, ACE2, via 2D diffusion [59, 60]. Coarse‐grained simulations and biophysical studies show that spike trimers cooperatively bind multiple ACE2 dimers, with a dominant binding mode in which several ACE2 dimers engage a single spike trimer [56]. Heparan sulfate, a SARS‐CoV‐2 attachment receptor, has been demonstrated to promote an allosteric conformational shift in spike‐bound ACE2, allowing a conserved linker region to facilitate ACE2 clustering to concentrate spike at the fusion site [57]. The biological relevance of this clustering is that it increases avidity, prolongs residence time, and makes fusion more likely by pushing spike into fusion‐competent states. This means infectivity is influenced not only by how tightly a single RBD binds ACE2, but also by how receptors and spikes are arranged on membranes [6, 55, 61].

Overall, the experimental systems used here isolate the intrinsic conformational dynamics and receptor‐binding behavior of individual spike trimers under well‐controlled conditions. While membrane organization and viral architecture will further modulate these processes in vivo, the relative differences observed between variants provide mechanistic insight into how evolutionary pressures may have shaped variant‐specific adaptations to simultaneously evade immunity, maintain or enhance receptor engagement, and fine‐tune the mechanical steps of viral entry—insights that are central to forecasting evolution and designing next‐generation countermeasures.

## 4 Methods

### 4.1 Genetic Constructs

The SARS‐CoV‐2 spike protein coding sequence was derived from the ancestral Wuhan‐1 isolate (NCBI Reference sequence: YP_009724390.1). Trimeric, pre‐fusion‐stabilized spike ectodomains (aa Q14–P1213, Wuhan‐1 numbering) of the ancestral SARS‐CoV‐2 spike and variants of concern Alpha (Genbank: QWE88920.1), Beta (QRN78347.1), Gamma (QVE55289.1), Delta (QWK65230.1), Omicron BA1, Omicron BA2 and Omicron BA5 were expressed with a C‐terminal T4 foldon trimerization domain, followed by a GGSSTG‐linker and an octa‐histidin tag from a mammalian expression vector (National Research Council, NRC, Ottawa, Canada). The prefusion‐stabilized ectodomain of all constructs is based on the spike HexaPro design [17], which has proline substitutions at positions F817P, A899P, A942P, K986P, and V987P (Wuhan‐1 numbering) and the polybasic cleavage site (682‐RRAR‐685) replaced by GSAS. Trimeric, prefusion‐stabilized spike ectodomains of Omicron XBB1.5 (QHD43416.1) was obtained from Acro Biosystems (Newark, DE; # SPN‐C524i‐50ug) and Omicron JN.1 (YP_009724390.1) was obtained from Sino Biological (Beijing, CN; # 40589‐V08H59) and had a similar genetic design, apart from the protease cleavage site being changed to 682‐RAAA‐685. The mutation profile of all variants used in this study is indicated relative to the wildtype sequence in Figure 1. All sequences had been codon‐optimized for expression in mammalian cells, were verified by Sanger sequencing (Microsynth, Balgach, CH), and are available upon request.

### 4.2 Recombinant Expression in HEK293‐6E Cells

Recombinant trimeric spike ectodomains were expressed in HEK293‐EBNA1‐6E (RRID: CVCL_HF20) suspension cells (licensed from National Research Council, NRC, Ottawa, Canada), as previously described [8, 62].

### 4.3 Protein Purification

Filtrated expression supernatants were about ten‐fold concentrated using a 0.005 m^2^ Pellicon XL‐Ultrafiltration module Biomax with a 50 kDa cut‐off (# PXB050A50, Merck Millipore, Burlington, MA) on a Labscale TFF System (Merck Millipore, Burlington, MA). Then, the buffer was exchanged to immobilized metal affinity chromatography (IMAC) binding buffer (20 mm phosphate buffer, pH 7.4, 500 mm NaCl). Before chromatography, the concentrated and diafiltrated supernatant was supplemented with 20 mm imidazole (final concentration) and sterile‐filtered. Proteins were further purified using a HisTrap HP 1‐mL column on an Äkta Pure system (both Cytiva, Marlborough, MA) and eluted in a linear gradient of 0%–100% buffer B (20 mm phosphate buffer pH 7.4, 500 mm NaCl, 500 mm imidazole) within 20 column volumes (CV). Elution fractions containing protein of high purity were pooled, concentrated to about 1 mg/mL and the buffer exchanged to gel filtration running buffer (50 mm Tris buffer pH 8.0, 200 mm NaCl, 300 mm L‐Arg). Proteins were polished by gel filtration using a HiLoad 16/600 Superose 6 prep column (Cytiva, Marlborough, MA). Fractions of the peak corresponding to the spike trimer were pooled and concentrated to 1 mg/mL using Amicon Ultra‐15 centrifugal filters with a 100 kDa cutoff (# UFC910024, Merck Millipore, Burlington, MA). Protein concentration was determined by measuring the absorbance at A280 on a NanoDrop 1000 spectrophotometer (Thermo Fisher Scientific, Waltham, MA) using extinction coefficients estimated from the respective amino acid sequences with the Expasy ProtParam tool. Protein sequences of purified proteins were confirmed by LC‐ESI‐MS/MS analysis and protein aliquots were stored at −80°C until further use.

### 4.4 Analytical Size‐Exclusion Chromatography

Recombinant spike proteins (100 µg, 1 mg/mL) were evaluated regarding purity and molecular weight distribution on a Shimadzu LC‐20A Prominence system equipped with a photodiode array (PDA) detector (Shimadzu), a refractive index detector (Shimadzu), and a MALS detector (miniDAWN TREOS, 3‐angle, Wyatt) on a Superose 6 Increase 10/300 GL (Cytiva, Marlborough, MA) column. Analytical size exclusion chromatography runs were performed at a constant flow rate of 0.75 mL/min with 50 mm Tris buffer pH 8.0, 100 mm L‐Arg, 150 mm NaCl, or 20 mm phosphate buffer pH 7.4 as mobile phase. For column calibration, a gel filtration standard (#1511901, Bio‐Rad, Hercules, CA) containing molecular weight markers between 1.35 and 670 kDa was used. Monomeric bovine serum albumin (#A1900 Sigma, St. Louis, MI) served as a molecular weight standard to normalize the instrument. Commercially obtained Omicron XBB1.5 and JN.1 spikes were verified to be trimeric by SEC‐MALS analysis according to the supplier.

### 4.5 Total Glycan Analysis using LC‐ESI‐MS

Glycans of wt peak 1 and 2 were analysed using LC‐ESI‐MS as described in [63].

### 4.6 Deglycosylation and Furin Cleavage of Recombinant Proteins

The purified variants were analyzed by Sodium dodecyl sulfate‐polyacrylamide gel electrophoresis (SDS‐PAGE) under reducing conditions. One µg of each purified protein and a molecular weight ladder (#26617, Thermo Fisher Scientific, Waltham, MA) was loaded on 4%–12% Bis‐Tris gradient gels (NuPAGE, Thermo Fisher Scientific, Waltham, MA), run in MOPS buffer at 200 V for 42 min, fixed and stained with GelCode Blue Safe Protein Stain (#1860957, Thermo Fisher Scientific, Waltham, MA). Proteins were further deglycosylated using PNGase (# P0704S, New England Biolabs, Ipswich, MA) or EndoHf (# P0703S, New England Biolabs, Ipswich, MA) for the removal of almost all *N*‐linked oligosaccharides from glycoproteins or only high‐mannose and some hybrid oligosaccharides, respectively. Deglycosylation of the proteins was performed according to the manufacturer's protocols. Briefly, proteins were denatured in a glycoprotein‐denaturing buffer at 100°C for 10 min, followed by deglycosylation at 37°C for 1 h and analysis by SDS‐PAGE. The absence of proteolytic cleavage at the mutated polybasic cleavage site was confirmed in a furin cleavage assay as per the manufacturer's instructions. Briefly, recombinant protein was incubated with recombinant furin (# P8077S, New England Biolabs, Ipswich, MA) for 4 h at 25°C and directly analyzed by SDS‐PAGE. A recombinant trimeric Wuhan‐1 spike protein with an intact polybasic protease cleavage site (# 40589‐V08B1, Sino Biologicals) served as a positive control for the assay.

### 4.7 High‐Speed AFM Sample Preparation and Imaging

Purified HexaPro SARS‐CoV‐2 Spike trimer variant stocks (Wuhan‐1, α, β, γ, δ; Omicron BA1, BA2, BA5, XBB1.5, JN.1) were diluted to 50 nM (10 mm Hepes pH 7.4, 140 mm NaCl, 5 mm KCl, 1 mm CaCl_2_, 1 mm MgCl_2_, 4.2 µm NiCl_2_). 1.5 µl of the Spike protein solution was incubated for 3 min on a freshly cleaved mica disc (1.5 mm diameter). The sample was washed 10 times with 2 µl imaging buffer (10 mm Hepes pH 7.4, 140 mm NaCl, 5 mm KCl, 1 mm CaCl_2_, 1 mm MgCl_2_) while keeping the surface moist. Measurement was conducted on a high‐speed AFM (HS‐AFM) [64] at room temperature (22°C). Ultra‐short cantilevers with a nominal spring constant of 0.15 N/m and a resonance frequency of 500 kHz (USCF1.2‐k0.15, Nanoworld AG) were used. Free amplitudes were ∼3 nm and the amplitude setpoint was 85%–90%. The imaging speed was 159.60 ms with an imaging area of 80 × 80 nm and 100 × 100 pixel for each video recorded. Spike trimers in the downward orientation (as described by Zhu et al., [8], where the stalk is facing upward and the three RBDs are in contact with the mica surface, were selected for further analysis [8, 24].

### 4.8 Image Processing, RBD Tracking and Analysis of Spike Dynamics

A Gaussian filter (σ = 0.6) was applied to the HS‐AFM images to reduce noise and improve visualization, using the Kodec 4.4.7.39 (Copyright (C) 2006–2008 N. Kodera, M. Sakashita, M. Imai, Kanazawa University, Biophysics Laboratory) software. Cartesian coordinates of the three RBDs and the Spike center were obtained by manually tracking their positions in each frame, using the Manual Tracking plugin (https://github.com/fiji/Manual_Tracking) of ImageJ 1.53t [65]. For RBD tracking, the point farthest away from the center was chosen. The positions of the three RBDs were normalized to the spike center position of each frame to exclude artifacts caused by drifting of the image frame and occasional movement of the particle itself. Normalized Cartesian coordinates were used to calculate the RBD's distance from the center, the distance between neighbouring RBDs, the displacement speed, and the angular displacement as described by Zhu et al., 2022 [8]. The distance from the center (RBD extension) (R) of the point of the RBD farthest away from the center was calculated in each frame from the normalized Cartesian coordinates (x_norm_, y_norm_), where the center is set to (0|0), with:

$$R=\sqrt{x_{norm}^{2}+y_{norm}^{2}}$$

The distance between neighbouring RBDs, D_RBDa−b_, was calculated from the normalized Cartesian coordinates, where a marks one RBD and b is the neighbouring RBD. Distances between RBD1‐RBD2, RBD2‐RBD3, and RBD3‐1 were calculated as followed:

$$D_{RBDa-b}=\sqrt{{\left(x_{norm-RBDa}-x_{norm-RBDb}\right)}^{2}+{\left(y_{norm-RBDa}-y_{norm-RBDb}\right)}^{2}\;}$$

The displacement speed (DS) was calculated from the distance one RBD travels between two subsequent frames (D_t_).

$$D_{t}=\sqrt{{\left({x_{norm}}_{t_{n+1}}-x_{{x_{norm}}_{t_{n}}}\right)}^{2}+{\left({y_{norm}}_{t_{n+1}}-y_{nor{m_{t}}_{n}}\right)}^{2}}$$

$$DS=\frac{D_{t}}{t_{n+1}-t_{n}}$$

The angular displacement(θ_d_) of the RBDs between two frames was calculated with:

$$\theta_{d}=\arccos\frac{x_{norm-t}*x_{norm-t+1}+y_{norm-t}*y_{norm-t+1}}{\sqrt{x_{norm+t}^{2}}+y_{norm+t}*\sqrt{x_{norm-t+1}^{2}}+y_{norm-t+1}}$$

### 4.9 Data Display and Statistical Analysis

The OriginPro (2018G) software was used to plot and analyse the data. A probability density function was generated using an in‐house Python script. An uncertainty of 0.8 nm was used to generate the probability density functions, as the pixel size is 0.8 nm. The data were checked for normal distribution using the D'Agostino K^2^ test (α = 0.05) for normal distribution. The datasets of the tested populations were almost exclusively found to be not normally distributed. Therefore, a Kruskal–Wallis ANOVA was conducted and showed that at the 0.001 level, the populations are significantly different (χ^2^ = 6571,96; DF = 11). To investigate statistical significance between populations, the post‐hoc Dunn's test was conducted. For a visual display of the results, heatmaps were generated from the mean rank differences of the Dunn's tests, using an in‐house Python script. To visually display the traces of the RBD movements (normalized to the movement of the spike center of mass) in Figure 2A, an in‐house Python script was used. For each variant, video recordings of 3 individual spike proteins with their 3 RBDs were analysed. The data of 3 spike x 3 RBDs per variant was pooled and analyzed, resulting in the following sample size: N(Wuhan‐1 peak 1) = 2319, N(Wuhan‐1 peak 2) = 1863, N(ɑ) = 3855, N(β) = 2697, N(γ peak 1) = 2127, N(γ peak 2) = 4287, N(δ) = 1335, N(o BA1) = 5190, N(o BA2) = 4839, N(o BA5) = 3516, N(o XBB1.5) = 1482), N(o JN.1) = 3117.

### 4.10 Functionalization of AFM Cantilever Tips with SARS‐CoV‐2 Spike Trimer

As previously described [8], AFM cantilever tips were conjugated with NHS‐PEG‐maleimide and thiol‐trisNTA. For the final protein conjugation step, cantilevers were incubated in 125–250 µg/mL His‐tagged Spike trimer in HBS at room temperature for 1 h and at 4°C for overnight. The solution contained additionally 200 µm NiCl_2_. Cantilevers were then stored at 4°C and washed 3 times in measurement solution before measurements.

### 4.11 Functionalization of AFM Cantilever with Inactivated SARS‐CoV‐2 Virus

The whole, inactivated SARS‐CoV‐2 virus (Wuhan‐1 strain) was conjugated to tipless AFM cantilevers and measured on Vero E6 cells as described in detail in [54].

### 4.12 Cells

Vero E6 cells (RRID: CVCL_0574) were provided by Josef Penninger (Institute of Molecular Biotechnology of the Austrian Academy of Sciences (IMBA), original source: ATCC CRL‐1586). Cells were cultured and prepared for the AFM measurements as previously described [8].

### 4.13 SMFS Measurements

All force‐distance curves were recorded at room temperature using a PicoPlus 5500 AFM setup with the software PicoScan 5 (Agilent Technologies, Chandler, AZ, USA) on living cells as previously described [8].

### 4.14 Force Data Analysis

For the force data analysis, MATLAB R2013a and OriginPro 9.0 were used. A one‐tailed t‐test was performed for statistical analysis. The Bell‐Evans theory [66] was employed to fit the width of the energy barrier x_B_ and the dissociation rate constant *k*_off_ for the obtained data of unbinding force vs. force loading rate. The force of multiple bonds was calculated from the William's Markov binding model [67], which assumes uncorrelated bond dissociation without mechanical coupling between individual bonds. To acquire the kinetic on‐rate *k*_on_, the measured data of binding activity vs. contact time were fitted as previously described [68].

## Author Contributions

**Sarah Stainer**, **Miriam Klausberger**, and **Peter Hinterdorfer**: Conceptualization; **Sarah Stainer**, **Rong Zhu**, **Manuel Reithofer**, **Katharina Stadlbauer**, **Gerhard Stadlmayr**, **Panchika Prangkio**, **Christoph Rippel**, **Markus Hahn**, **Yoo Jin Oh**, **Miriam Klausberger**, and **Peter Hinterdorfer**: Data Curation; **Sarah Stainer**, **Rong Zhu**, **Manuel Reithofer**, **Katharina Stadlbauer**, **Gerhard Stadlmayr**, **Panchika Prangkio**, **Christoph Rippel**, **Markus Hahn**, **Yoo Jin Oh**, **Miriam Klausberger**, and **Peter Hinterdorfer**: Formal Analysis; **Miriam Klausberger**, **Daniel Ruzek**, **Robert Tampé**, and **Peter Hinterdorfer**: Funding Acquisition; **Sarah Stainer**, **Rong Zhu**, **Manuel Reithofer**, **Katharina Stadlbauer**, **Gerhard Stadlmayr**, **Panchika Prangkio**, **Christoph Rippel**, **Markus Hahn**, **Aniko Eva Kasztner**, **Stefanie Stohl**, **Petr Bednar**, **Daniel Ruzek**, **Yoo Jin Oh**, **Miriam Klausberger**, and **Peter Hinterdorfer**: Investigation; **Sarah Stainer**, **Rong Zhu**, **Manuel Reithofer**, **Katharina Stadlbauer**, **Gerhard Stadlmayr**, **Panchika Prangkio**, **Christoph Rippel**, **Markus Hahn**, **Aniko Eva Kasztner**, **Stefanie Stohl**, **Petr Bednar**, **Daniel Ruzek**, **David Glück**, **Robert Tampé**, **Yoo Jin Oh**, **Miriam Klausberger**, and **Peter Hinterdorfer**: Methodology; **Miriam Klausberger** and **Peter Hinterdorfer**: Project Administration; **Miriam Klausberger**, **Petr Bednar**, **Daniel Ruzek**, **David Glück**, **Robert Tampé**, **Gordana Wozniak‐Knopp**, and **Peter Hinterdorfer**: Resources; **Sarah Stainer**: Software; **Miriam Klausberger** and **Peter Hinterdorfer**: Supervision; **All Authors**: Validation; **Sarah Stainer**, **Rong Zhu**, **Panchika Prangkio**, **Christoph Rippel**, **Markus Hahn**, **Yoo Jin Oh**, **Miriam Klausberger**, and **Peter Hinterdorfer**: Visualization; **Stefanie Stohl**, **Rong Zhu**, **Manuel Reithofer**, **Katharina Stadlbauer**, **Gerhard Stadlmayr**, **Miriam Klausberger**, and **Peter Hinterdorfer**: Writing – original draft; **All Authors**: Writing, review & editing .

## Conflicts of Interest

The authors declare no conflicts of interest.

## Supporting information




**Supporting File 1**: advs77290‐sup‐0001‐SuppMat.docx.

**Supporting File 2**: advs77290‐sup‐0002‐MovieS1.avi.

**Supporting File 3**: advs77290‐sup‐0003‐MovieS2.avi.

**Supporting File 4**: advs77290‐sup‐0004‐MovieS3.avi.

**Supporting File 5**: advs77290‐sup‐0005‐MovieS4.avi.

**Supporting File 6**: advs77290‐sup‐0006‐MovieS5.avi.

**Supporting File 7**: advs77290‐sup‐0007‐MovieS6.avi.

**Supporting File 8**: advs77290‐sup‐0008‐MovieS7.avi.

**Supporting File 9**: advs77290‐sup‐0009‐MovieS8.avi.

**Supporting File 10**: advs77290‐sup‐0010‐MovieS9.avi.

**Supporting File 11**: advs77290‐sup‐0011‐MovieS10.avi.

**Supporting File 12**: advs77290‐sup‐0012‐MovieS11.avi.

**Supporting File 13**: advs77290‐sup‐0013‐MovieS12.avi.

## Data Availability

The data that support the findings of this study are available from the corresponding author upon reasonable request.
