# Supplementary material for: Switching Spike Plasticity Shapes ACE2 Engagement Across SARS‐CoV‐2 Variants
Source: Adv Sci (Weinh). 2026 Aug 31:e77290. Online ahead of print. doi: 10.1002/advs.77290 (PMC13528683; doi:10.1002/advs.77290)
Supplement: Supplementary file 1 [file ADVS-9999-e77290-s010.docx]

**Content**

**Figure S1-8**

**Table S1-8**

**Figure S1:** **Both fractions of the resolved double peak correspond to the molecular weight of the SARS-CoV-2 spike protein.** HPLC-SEC-MALS profiles of the SEC-fractionated faster-eluting (black) and slower-eluting (petrol) species of the Wuhan-1 double-peak. LS - light scattering.

**Figure S2:** **Differences in RBD Displacement Speed and RBD Angular Displacement of the different Spike Variants.** **(A)** Displacement speed of the RBDs portrayed as boxplots, with individual datapoints displayed in scatterplots and overlayed with their probability density functions. **(B)** Heatmap of the displacement speed created from the post hoc Dunn’s test and the resulting mean rank differences. Mean rank differences gained from pairwise comparisons of the post hoc Dunn’s test^42^ were plotted in a 2-D false color map, whereby negative (blue-shaded) mean rank differences indicate that spike variants displayed on the x-axis have a significantly smaller RBD extension compared to the variants on the y-axis, while red colours indicate positive mean rank differences and a larger RBD extensions on variants on the x-axis compared to the y-axis. The false color scale bar represents the mean rank differences. n.s. indicates that the compared datasets are not significantly different (ɑ=0.001). **(C)** Angular Displacement of the RBDs portrayed as boxplots, with individual datapoints displayed in scatterplots and overlayed with their probability density functions. **(D)** Heatmap of the angular displacement created from the post hoc Dunn’s test and the resulting mean rank differences. (N(Wuhan-1 peak 1)=2319, N(Wuhan-1 peak 2)=1863, N(ɑ)=3855, N(ß)=2697, N(γ peak 1)=2127, N(γ peak 2)=4287, N(δ)=1335, N(o BA1)=5190, N(o BA2)=4839, N(o BA5)=3516, N(o XBB1.5)=1482), N(o JN.1) = 3117)

**Figure S3:** **Graphical correlation of RBD distance from the center of mass and the distance between the RBDs.** Linear regression (R = 0.88, p = 0.0002) of the mean RBD distances from the center and the mean distances between the RBDs. N(Wuhan-1 peak 1)=2319, N(Wuhan-1 peak 2)=1863, N(ɑ)=3855, N(ꞵ)=2697, N(γ peak 1)=2127, N(γ peak 2)=4287, N(δ)=1335, N(o BA1)=5190, N(o BA2)=4839, N(o BA5)=3516, N(o XBB1.5)=1482), N(o JN.1) = 3117.

**Figure S4: Binding activity *versus* contact time measured by each variant.**

**Figure S5: Percentage of multiple bonds *versus* contact time measured by each variant.**

**Figure S6: Unbinding force *versus* force loading rate measured by each variant.** The green curve is the fitting with Bell-Evans’ model. The orange or blue curve is the calculation result according to William’s two or three bonds model.

**Figure S7: Correlation coefficients between parameters from all variants studied in this work (including W1, W2,** ɑ**,** ꞵ**, 𝛾1, 𝛾2, 𝛿, 𝜊BA1, 𝜊BA2, 𝜊BA5, 𝜊XBB1.5, 𝜊JN.1).** Mean values of k_on_, k_off_, k_D_, k_2_, P_2B_, P_3B_, D_C_, D_R_, V, and A from each variant are used to calculate the Spearman correlation coefficient. Two-sided test of significance is used. *: correlation is significant at the 0.05 level. **: correlation is significant at the 0.01 level. ***: correlation is significant at the 0.001 level. P_2B_: percentage of 2-bonds. P_3B_: percentage of 3-bonds. D_C_: distance of RBD extension from center. D_R_: distance between RBDs. V: displacement speed. A: angular displacement of RBD.

**Figure S8: The inactivated whole virus (Wuhan-1 strain) shows comparable forces to engineered soluble, trimeric spike ectodomain (Wuhan-1, pooled).**

**Table S1: Total glycan analysis of the SEC-resolved fraction of the Wuhan-1 double peak.** Proportion of glycoforms in % assessed by LC-ESI-MS.

|  | **Wuhan-1** | |
| --- | --- | --- |
| **Glycan** | **Peak Front** | **Peak Tail** |
| H2N2 | 0.33% | 0.18% |
| H3N2 | 0.55% | 0.33% |
| H3N2F | 0.18% | 0.28% |
| H4N2 | 1.75% | 1.55% |
| H3N3 | 1.65% | 1.01% |
| H5N2 | 13.31% | 13.64% |
| H3N3F1 | 2.64% | 2.76% |
| H4N3 | 2.16% | 1.28% |
| H3N4 | 1.93% | 1.18% |
| H6N2 | 8.33% | 4.06% |
| H4N3F1 | 1.75% | 1.85% |
| H5N3 | 4.91% | 2.99% |
| H3N4F1 | 5.59% | 4.83% |
| H4N4 | 0.84% | 0.62% |
| H7N2 | 5.80% | 2.89% |
| H6N3 | 1.84% | 2.14% |
| H4N4F1 | 2.45% | 3.51% |
| H5N4 | 1.65% | 2.05% |
| H3N5F1 | 9.61% | 8.66% |
| H4N3SF1 | 0.12% | 0.23% |
| H8N2 | 2.87% | 1.40% |
| H5N4F1 | 3.04% | 4.03% |
| H6N4 | 0.01% | 0.01% |
| H4N5 | 1.00% | 0.88% |
| H3N6F1 | 5.66% | 5.46% |
| H4N4S1F1 | 1.18% | 2.36% |
| H5N4S1 | 1.10% | 1.70% |
| H4N5FF | 0.86% | 1.28% |
| H5N5F1 | 2.80% | 3.04% |
| H6N5 | 1.74% | 1.96% |
| H5N4S1F1 | 3.36% | 6.20% |
| H4N5S1F1 | 1.20% | 2.30% |
| H6N5F1 | 1.86% | 2.61% |
| H3N6F1S1 | 0.12% | 0.24% |
| H5N5S1F1 | 0.72% | 1.24% |
| H6N5S1 | 0.52% | 1.00% |
| H6N5S1F1 | 1.26% | 2.46% |
| H7N6F1 | 1.17% | 1.92% |
| H6N5S2 | 0.08% | 0.17% |
| H7N6S1F1 | 2.05% | 3.70% |

**Table S2: RBD displacement speed descriptive statistics parameters.**

N is the number of observations (gained from 3 spike proteins with data from 3 RBDs each), x is the arithmetic mean, STD is the standard deviation, Min. and Max. are the minimum and maximum value of the observed population, respectively, P25 and P75 the 25th and 75th percentile of the population, respectively.

| Variant | N | x [nm/s] | STD [nm/s] | Minimum | Median | Maximum | P25 | P75 |
| --- | --- | --- | --- | --- | --- | --- | --- | --- |
| W1 | 2310 | 48,12 | 39,87 | 0 | 36,28 | 279,10 | 18,14 | 68,44 |
| W2 | 1854 | 48,99 | 41,51 | 0 | 36,28 | 264,81 | 18,14 | 67,13 |
| α | 3846 | 42,48 | 32,60 | 0 | 33,75 | 212,28 | 20,13 | 55,57 |
| β | 2688 | 43,64 | 36,18 | 0 | 32,22 | 232,81 | 15,91 | 61,42 |
| γ1 | 2118 | 41,19 | 35,58 | 0 | 30,61 | 261,83 | 14,23 | 60,59 |
| γ2 | 4278 | 37,15 | 34,24 | 0 | 25,66 | 331,01 | 11,25 | 52,53 |
| δ | 1326 | 31,02 | 25,40 | 0 | 25,16 | 178,46 | 11,25 | 41,49 |
| o BA1 | 5181 | 30,54 | 28,07 | 0 | 21,35 | 235,83 | 11,25 | 41,49 |
| o BA2 | 4830 | 36,60 | 29,72 | 0 | 28,46 | 199,04 | 15,09 | 50,57 |
| o BA5 | 3507 | 37,76 | 31,33 | 0 | 27,10 | 209,40 | 15,09 | 51,80 |
| o XBB1.5 | 1473 | 23,52 | 24,52 | 0 | 15,91 | 171,07 | 7,12 | 30,61 |
| o JN.1 | 3108 | 24,25 | 21,19 | 0 | 18,14 | 166,34 | 10,06 | 31,82 |

**Table S3 RBD displacement speed- Statistical test parameters and results of the Post-hoc Dunn’s test.**

Sig = 1 indicates that the difference of the means is significant at the 0.001 level

Sig = 0 indicates that the difference of the means is NOT significant at the 0.001 level

| Comparison | Mean Rank Diff | Z | P-value | Sig |
| --- | --- | --- | --- | --- |
| "WT 1" "WT 2" | -170,73 | -0,52 | 1 | 0 |
| "WT 1" "α" | 522,30 | 1,88 | 1 | 0 |
| "WT 1" "β" | 1.110,73 | 3,71 | 0,01349 | 0 |
| "WT 1" "γ 1" | 1.990,54 | 6,28 | 2,29E-08 | 1 |
| "WT 1" "γ 2" | 3.468,16 | 12,74 | 2,30E-35 | 1 |
| "WT 1" "δ" | 4.524,49 | 12,46 | 8,50E-34 | 1 |
| "WT 1" "BA1" | 5.333,44 | 20,22 | 4,18E-89 | 1 |
| "WT 1" "BA2" | 2.786,51 | 10,45 | 9,83E-24 | 1 |
| "WT 1" "BA5" | 2.506,96 | 8,87 | 4,64E-17 | 1 |
| "WT 1" "XBB1.5" | 8.426,53 | 23,97 | 3,62E-125 | 1 |
| "WT 1" "JN.1" | 7.357,24 | 25,40 | 1,50E-140 | 1 |
| "WT 2" "α" | 693,04 | 2,33 | 1 | 0 |
| "WT 2" "β" | 1.281,46 | 4,03 | 0,00374 | 0 |
| "WT 2" "γ 1" | 2.161,27 | 6,45 | 7,58E-09 | 1 |
| "WT 2" "γ 2" | 3.638,90 | 12,41 | 1,45E-33 | 1 |
| "WT 2" "δ" | 4.695,23 | 12,38 | 2,13E-33 | 1 |
| "WT 2" "BA1" | 5.504,17 | 19,29 | 4,12E-81 | 1 |
| "WT 2" "BA2" | 2.957,24 | 10,27 | 6,53E-23 | 1 |
| "WT 2" "BA5" | 2.677,70 | 8,85 | 6,01E-17 | 1 |
| "WT 2" "XBB1.5" | 8.597,27 | 23,36 | 6,51E-119 | 1 |
| "WT 2" "JN.1" | 7.527,97 | 24,33 | 5,70E-129 | 1 |
| "α" "β" | 588,42 | 2,22 | 1 | 0 |
| "α" "γ 1" | 1.468,23 | 5,15 | 1,75E-05 | 1 |
| "α" "γ 2" | 2.945,86 | 12,58 | 1,91E-34 | 1 |
| "α" "δ" | 4.002,19 | 11,92 | 6,08E-31 | 1 |
| "α" "BA1" | 4.811,13 | 21,44 | 3,64E-100 | 1 |
| "α" "BA2" | 2.264,20 | 9,94 | 1,88E-21 | 1 |
| "α" "BA5" | 1.984,66 | 8,06 | 4,92E-14 | 1 |
| "α" "XBB1.5" | 7.904,23 | 24,47 | 2,10E-130 | 1 |
| "α" "JN.1" | 6.834,94 | 26,88 | 2,50E-157 | 1 |
| "β" "γ 1" | 879,81 | 2,87 | 0,2689 | 0 |
| "β" "γ 2" | 2.357,44 | 9,09 | 6,82E-18 | 1 |
| "β" "δ" | 3.413,77 | 9,65 | 3,27E-20 | 1 |
| "β" "BA1" | 4.222,71 | 16,85 | 6,86E-62 | 1 |
| "β" "BA2" | 1.675,78 | 6,61 | 2,61E-09 | 1 |
| "β" "BA5" | 1.396,24 | 5,17 | 1,58E-05 | 1 |
| "β" "XBB1.5" | 7.315,81 | 21,41 | 7,70E-100 | 1 |
| "β" "JN.1" | 6.246,52 | 22,50 | 3,05E-110 | 1 |
| "γ 1" "γ 2" | 1.477,62 | 5,28 | 8,74E-06 | 1 |
| "γ 1" "δ" | 2.533,95 | 6,86 | 4,43E-10 | 1 |
| "γ 1" "BA1" | 3.342,90 | 12,29 | 6,37E-33 | 1 |
| "γ 1" "BA2" | 795,97 | 2,90 | 0,24857 | 0 |
| "γ 1" "BA5" | 516,43 | 1,78 | 1 | 0 |
| "γ 1" "XBB1.5" | 6.435,99 | 17,99 | 1,43E-70 | 1 |
| "γ 1" "JN.1" | 5.366,70 | 18,07 | 3,82E-71 | 1 |
| "γ 2" "δ" | 1.056,33 | 3,19 | 0,09459 | 0 |
| "γ 2" "BA1" | 1.865,27 | 8,56 | 7,16E-16 | 1 |
| "γ 2" "BA2" | -681,66 | -3,08 | 0,13675 | 0 |
| "γ 2" "BA5" | -961,20 | -4,00 | 0,00414 | 0 |
| "γ 2" "XBB1.5" | 4.958,37 | 15,57 | 7,85E-53 | 1 |
| "γ 2" "JN.1" | 3.889,08 | 15,65 | 2,13E-53 | 1 |
| "δ" "BA1" | 808,94 | 2,49 | 0,83539 | 0 |
| "δ" "BA2" | -1.737,99 | -5,32 | 6,94E-06 | 1 |
| "δ" "BA5" | -2.017,53 | -5,94 | 1,92E-07 | 1 |
| "δ" "XBB1.5" | 3.902,04 | 9,78 | 9,29E-21 | 1 |
| "δ" "JN.1" | 2.832,75 | 8,19 | 1,70E-14 | 1 |
| "BA1" "BA2" | -2.546,93 | -12,08 | 9,03E-32 | 1 |
| "BA1" "BA5" | -2.826,47 | -12,26 | 9,70E-33 | 1 |
| "BA1" "XBB1.5" | 3.093,10 | 9,94 | 1,91E-21 | 1 |
| "BA1" "JN.1" | 2.023,80 | 8,46 | 1,75E-15 | 1 |
| "BA2" "BA5" | -279,54 | -1,20 | 1 | 0 |
| "BA2" "XBB1.5" | 5.640,03 | 17,97 | 2,06E-70 | 1 |
| "BA2" "JN.1" | 4.570,74 | 18,85 | 1,79E-77 | 1 |
| "BA5" "XBB1.5" | 5.919,57 | 18,08 | 2,79E-71 | 1 |
| "BA5" "JN.1" | 4.850,28 | 18,68 | 5,17E-76 | 1 |
| "XBB1.5" "JN.1" | -1.069,29 | -3,21 | 0,0887 | 0 |

**Table S4: RBD angular displacement descriptive statistics parameters.**

N is the number of observations (gained from 3 spike proteins with data from 3 RBDs each), x is the arithmetic mean, STD is the standard deviation, Min. and Max. are the minimum and maximum value of the observed population, respectively, P25 and P75 the 25th and 75th percentile of the population, respectively.

| Variant | N | x  [degree] | STD [degree] | Min. [degree] | Median [degree] | Max. [degree] | P25 [degree] | P75 [degree] |
| --- | --- | --- | --- | --- | --- | --- | --- | --- |
| WT 1 | 2310 | 19,59 | 19,64 | 0 | 13,09 | 159,78 | 4,99 | 27,78 |
| WT 2 | 1854 | 19,95 | 20,62 | 0 | 13,52 | 171,71 | 5,43 | 27,53 |
| α | 3846 | 17,26 | 16,02 | 0 | 12,81 | 121,39 | 5,84 | 23,50 |
| β | 2688 | 16,97 | 16,82 | 0 | 11,24 | 114,86 | 4,40 | 24,57 |
| γ 1 | 2118 | 16,25 | 16,73 | 0 | 10,68 | 128,43 | 4,16 | 24,10 |
| γ 2 | 4278 | 14,62 | 16,46 | 0 | 8,31 | 178,06 | 2,73 | 21,06 |
| δ | 1326 | 13,10 | 13,04 | 0 | 9,18 | 100,30 | 3,78 | 18,43 |
| BA1 | 5181 | 13,49 | 15,37 | 0 | 7,82 | 118,08 | 2,75 | 18,82 |
| BA2 | 4830 | 15,16 | 14,96 | 0 | 10,32 | 109,94 | 3,90 | 21,77 |
| BA5 | 3507 | 16,01 | 16,58 | 0 | 10,25 | 131,27 | 4,04 | 22,55 |
| XBB1.5 | 1473 | 9,71 | 11,61 | 0 | 5,71 | 86,73 | 2,17 | 12,91 |
| JN.1 | 3108 | 12,24 | 12,24 | 0 | 8,57 | 95,34 | 3,69 | 16,81 |

**Table S5: Angular displacement - Statistical test parameters and results of the Post-hoc Dunn’s test.**

| Comparison | Mean Rank Diff | Z | P-value | Sig |
| --- | --- | --- | --- | --- |
| "WT 1" "WT 2" | -210,20 | -0,64 | 1 | 0 |
| "WT 1" "α" | 220,22 | 0,79 | 1 | 0 |
| "WT 1" "β" | 1.163,94 | 3,89 | 0,00657 | 0 |
| "WT 1" "γ 1" | 1.661,93 | 5,24 | 1,06E-05 | 1 |
| "WT 1" "γ 2" | 3.327,58 | 12,22 | 1,51E-32 | 1 |
| "WT 1" "δ" | 3.178,08 | 8,75 | 1,41E-16 | 1 |
| "WT 1" "BA1" | 3.834,48 | 14,54 | 4,56E-46 | 1 |
| "WT 1" "BA2" | 2.080,46 | 7,80 | 4,05E-13 | 1 |
| "WT 1" "BA5" | 1.804,68 | 6,39 | 1,11E-08 | 1 |
| "WT 1" "XBB1.5" | 6.571,28 | 18,69 | 3,64E-76 | 1 |
| "WT 1" "JN.1" | 3.702,41 | 12,78 | 1,32E-35 | 1 |
| "WT 2" "α" | 430,42 | 1,44 | 1 | 0 |
| "WT 2" "β" | 1.374,14 | 4,32 | 0,00104 | 0 |
| "WT 2" "γ 1" | 1.872,13 | 5,58 | 1,55E-06 | 1 |
| "WT 2" "γ 2" | 3.537,78 | 12,07 | 1,02E-31 | 1 |
| "WT 2" "δ" | 3.388,28 | 8,94 | 2,65E-17 | 1 |
| "WT 2" "BA1" | 4.044,68 | 14,18 | 8,40E-44 | 1 |
| "WT 2" "BA2" | 2.290,66 | 7,95 | 1,20E-13 | 1 |
| "WT 2" "BA5" | 2.014,88 | 6,66 | 1,86E-09 | 1 |
| "WT 2" "XBB1.5" | 6.781,48 | 18,43 | 4,99E-74 | 1 |
| "WT 2" "JN.1" | 3.912,62 | 12,65 | 7,63E-35 | 1 |
| "α" "β" | 943,72 | 3,56 | 0,02441 | 0 |
| "α" "γ 1" | 1.441,71 | 5,05 | 2,85E-05 | 1 |
| "α" "γ 2" | 3.107,36 | 13,26 | 2,45E-38 | 1 |
| "α" "δ" | 2.957,86 | 8,81 | 8,23E-17 | 1 |
| "α" "BA1" | 3.614,26 | 16,11 | 1,49E-56 | 1 |
| "α" "BA2" | 1.860,24 | 8,17 | 2,12E-14 | 1 |
| "α" "BA5" | 1.584,46 | 6,44 | 8,04E-09 | 1 |
| "α" "XBB1.5" | 6.351,06 | 19,66 | 3,07E-84 | 1 |
| "α" "JN.1" | 3.482,19 | 13,69 | 7,22E-41 | 1 |
| "β" "γ 1" | 497,99 | 1,63 | 1 | 0 |
| "β" "γ 2" | 2.163,64 | 8,34 | 4,96E-15 | 1 |
| "β" "δ" | 2.014,14 | 5,69 | 8,23E-07 | 1 |
| "β" "BA1" | 2.670,53 | 10,66 | 1,07E-24 | 1 |
| "β" "BA2" | 916,52 | 3,61 | 0,01999 | 0 |
| "β" "BA5" | 640,73 | 2,37 | 1 | 0 |
| "β" "XBB1.5" | 5.407,34 | 15,82 | 1,44E-54 | 1 |
| "β" "JN.1" | 2.538,47 | 9,14 | 4,05E-18 | 1 |
| "γ 1" "γ 2" | 1.665,65 | 5,95 | 1,81E-07 | 1 |
| "γ 1" "δ" | 1.516,15 | 4,11 | 0,00265 | 0 |
| "γ 1" "BA1" | 2.172,55 | 7,99 | 8,87E-14 | 1 |
| "γ 1" "BA2" | 418,53 | 1,52 | 1 | 0 |
| "γ 1" "BA5" | 142,75 | 0,49 | 1 | 0 |
| "γ 1" "XBB1.5" | 4.909,35 | 13,73 | 4,67E-41 | 1 |
| "γ 1" "JN.1" | 2.040,48 | 6,87 | 4,25E-10 | 1 |
| "γ 2" "δ" | -149,50 | -0,45 | 1 | 0 |
| "γ 2" "BA1" | 506,90 | 2,33 | 1 | 0 |
| "γ 2" "BA2" | -1.247,12 | -5,63 | 1,16E-06 | 1 |
| "γ 2" "BA5" | -1.522,90 | -6,34 | 1,50E-08 | 1 |
| "γ 2" "XBB1.5" | 3.243,70 | 10,18 | 1,53E-22 | 1 |
| "γ 2" "JN.1" | 374,84 | 1,51 | 1 | 0 |
| "δ" "BA1" | 656,39 | 2,02 | 1 | 0 |
| "δ" "BA2" | -1.097,62 | -3,36 | 0,05176 | 0 |
| "δ" "BA5" | -1.373,40 | -4,04 | 0,00351 | 0 |
| "δ" "XBB1.5" | 3.393,20 | 8,50 | 1,22E-15 | 1 |
| "δ" "JN.1" | 524,33 | 1,52 | 1 | 0 |
| "BA1" "BA2" | -1.754,01 | -8,32 | 5,88E-15 | 1 |
| "BA1" "BA5" | -2.029,80 | -8,81 | 8,63E-17 | 1 |
| "BA1" "XBB1.5" | 2.736,81 | 8,79 | 9,71E-17 | 1 |
| "BA1" "JN.1" | -132,06 | -0,55 | 1 | 0 |
| "BA2" "BA5" | -275,79 | -1,18 | 1 | 0 |
| "BA2" "XBB1.5" | 4.490,82 | 14,31 | 1,22E-44 | 1 |
| "BA2" "JN.1" | 1.621,95 | 6,69 | 1,47E-09 | 1 |
| "BA5" "XBB1.5" | 4.766,60 | 14,56 | 3,22E-46 | 1 |
| "BA5" "JN.1" | 1.897,74 | 7,31 | 1,80E-11 | 1 |
| "XBB1.5" "JN.1" | -2.868,87 | -8,60 | 5,13E-16 | 1 |

**Table S6: RBD extension descriptive statistics parameters.**

N is the number of observations (gained from 3 spike proteins with data from 3 RBDs each), x is the arithmetic mean, STD is the standard deviation, Min. and Max. are the minimum and maximum value of the observed population, respectively, P25 and P75 the 25th and 75th percentile of the population, respectively.

| Variant | N | x [nm] | STD [nm] | Min. | Median | Max. | P25 | P75 |
| --- | --- | --- | --- | --- | --- | --- | --- | --- |
| W1 | 2319 | 20,05 | 4,21 | 9,12 | 19,68 | 39,36 | 17,23 | 22,66 |
| W2 | 1863 | 19,95 | 3,96 | 8,80 | 19,48 | 34,62 | 17,10 | 22,53 |
| α | 3855 | 20,72 | 3,42 | 10,67 | 20,49 | 40,26 | 18,26 | 22,91 |
| β | 2697 | 21,62 | 3,98 | 11,54 | 20,80 | 39,58 | 19,02 | 23,53 |
| γ1 | 2127 | 21,04 | 3,91 | 9,12 | 20,80 | 39,47 | 18,42 | 23,20 |
| γ2 | 4287 | 21,23 | 3,61 | 6,40 | 21,53 | 32,96 | 18,61 | 24,05 |
| δ | 1335 | 18,22 | 3,35 | 8,84 | 18,14 | 31,94 | 16,00 | 20,25 |
| o BA1 | 5190 | 18,35 | 3,49 | 7,38 | 18,42 | 30,79 | 16,02 | 20,80 |
| o BA2 | 4839 | 19,76 | 2,89 | 10,43 | 19,76 | 32,25 | 17,76 | 21,61 |
| o BA5 | 3516 | 19,75 | 3,70 | 8,62 | 19,53 | 32,18 | 17,23 | 22,46 |
| o XBB1.5 | 1482 | 18,62 | 3,13 | 11,11 | 18,42 | 28,28 | 16,20 | 20,94 |
| o JN1 | 3117 | 15,33 | 2,87 | 7,38 | 15,09 | 28,90 | 13,58 | 16,82 |

**Table S7: RBD extension - Statistical test parameters and results of the Post-hoc Dunn’s test.**

Sig = 1 indicates that the difference of the means is significant at the 0.001 level

Sig = 0 indicates that the difference of the means is NOT significant at the 0.001 level

| Comparison | Mean Rank Diff | Z | p-value | Sig |
| --- | --- | --- | --- | --- |
| W1" "W2 | 193,74 | 0,59 | 1 | 0 |
| W1" "α | -2.269,03 | -8,17 | 2,10E-14 | 1 |
| W1" "β | -3.922,00 | -13,10 | 2,23E-37 | 1 |
| W1" "γ 1 | -2.731,07 | -8,60 | 5,11E-16 | 1 |
| W1" "γ 2 | -3.850,64 | -14,13 | 1,69E-43 | 1 |
| W1" "δ | 4.773,90 | 13,14 | 1,25E-37 | 1 |
| W1" "BA1 | 4.090,53 | 15,49 | 2,74E-52 | 1 |
| W1" "BA2 | 203,55 | 0,76 | 1 | 0 |
| W1" "BA5 | 291,83 | 1,03 | 1 | 0 |
| W1" "XBB1.5 | 3.666,90 | 10,43 | 1,22E-23 | 1 |
| W1" "JN1 | 12.477,99 | 43,03 | 0 | 1 |
| W2" "α | -2.462,76 | -8,25 | 1,00E-14 | 1 |
| W2" "β | -4.115,74 | -12,92 | 2,26E-36 | 1 |
| W2" "γ 1 | -2.924,81 | -8,72 | 1,88E-16 | 1 |
| W2" "γ 2 | -4.044,38 | -13,78 | 2,09E-41 | 1 |
| W2" "δ | 4.580,16 | 12,08 | 8,86E-32 | 1 |
| W2" "BA1 | 3.896,79 | 13,65 | 1,41E-40 | 1 |
| W2" "BA2 | 9,82 | 0,03 | 1 | 0 |
| W2" "BA5 | 98,10 | 0,32 | 1 | 0 |
| W2" "XBB1.5 | 3.473,16 | 9,44 | 2,53E-19 | 1 |
| W2" "JN1 | 12.284,25 | 39,67 | 0 | 1 |
| α" "β | -1.652,97 | -6,23 | 3,13E-08 | 1 |
| α" "γ 1 | -462,04 | -1,62 | 1 | 0 |
| α" "γ 2 | -1.581,61 | -6,74 | 1,05E-09 | 1 |
| α" "δ | 7.042,93 | 20,98 | 7,27E-96 | 1 |
| α" "BA1 | 6.359,56 | 28,29 | 3,18E-174 | 1 |
| α" "BA2 | 2.472,58 | 10,83 | 1,60E-25 | 1 |
| α" "BA5 | 2.560,86 | 10,39 | 1,90E-23 | 1 |
| α" "XBB1.5 | 5.935,93 | 18,37 | 1,56E-73 | 1 |
| α" "JN1 | 14.747,01 | 57,90 | 0 | 1 |
| β" "γ 1 | 1.190,93 | 3,88 | 0,00678 | 0 |
| β" "γ 2 | 71,36 | 0,27 | 1 | 0 |
| β" "δ | 8.695,90 | 24,58 | 1,48E-131 | 1 |
| β" "BA1 | 8.012,53 | 31,92 | 8,04E-222 | 1 |
| β" "BA2 | 4.125,56 | 16,24 | 1,81E-57 | 1 |
| β" "BA5 | 4.213,84 | 15,57 | 7,72E-53 | 1 |
| β" "XBB1.5 | 7.588,90 | 22,20 | 2,42E-107 | 1 |
| β" "JN1 | 16.399,99 | 58,98 | 0 | 1 |
| γ 1" "γ 2 | -1.119,57 | -3,99 | 0,00432 | 0 |
| γ 1" "δ | 7.504,97 | 20,33 | 4,79E-90 | 1 |
| γ 1" "BA1 | 6.821,60 | 25,06 | 9,05E-137 | 1 |
| γ 1" "BA2 | 2.934,63 | 10,67 | 9,42E-25 | 1 |
| γ 1" "BA5 | 3.022,91 | 10,41 | 1,51E-23 | 1 |
| γ 1" "XBB1.5 | 6.397,97 | 17,88 | 1,05E-69 | 1 |
| γ 1" "JN1 | 15.209,06 | 51,15 | 0 | 1 |
| γ 2" "δ | 8.624,54 | 26,03 | 1,69E-147 | 1 |
| γ 2" "BA1 | 7.941,17 | 36,39 | 3,87E-288 | 1 |
| γ 2" "BA2 | 4.054,19 | 18,28 | 7,70E-73 | 1 |
| γ 2" "BA5 | 4.142,48 | 17,22 | 1,25E-64 | 1 |
| γ 2" "XBB1.5 | 7.517,54 | 23,59 | 2,89E-121 | 1 |
| γ 2" "JN1 | 16.328,63 | 65,61 | 0 | 1 |
| δ" "BA1 | -683,37 | -2,11 | 1 | 0 |
| δ" "BA2 | -4.570,34 | -13,98 | 1,32E-42 | 1 |
| δ" "BA5 | -4.482,06 | -13,19 | 6,97E-38 | 1 |
| δ" "XBB1.5 | -1.107,00 | -2,77 | 0,36474 | 0 |
| δ" "JN1 | 7.704,09 | 22,28 | 4,15E-108 | 1 |
| BA1" "BA2 | -3.886,97 | -18,40 | 9,27E-74 | 1 |
| BA1" "BA5 | -3.798,69 | -16,45 | 5,70E-59 | 1 |
| BA1" "XBB1.5 | -423,63 | -1,36 | 1 | 0 |
| BA1" "JN1 | 8.387,46 | 35,01 | 1,19E-266 | 1 |
| BA2" "BA5 | 88,28 | 0,38 | 1 | 0 |
| BA2" "XBB1.5 | 3.463,35 | 11,03 | 1,75E-26 | 1 |
| BA2" "JN1 | 12.274,43 | 50,55 | 0 | 1 |
| BA5" "XBB1.5 | 3.375,06 | 10,31 | 4,34E-23 | 1 |
| BA5" "JN1 | 12.186,15 | 46,85 | 0 | 1 |
| XBB1.5" "JN1 | 8.811,09 | 26,41 | 6,84E-152 | 1 |

**Table S8. Comparison of k_off_ of single bond ruptures from the engineered spike and the whole virus on Vero E6 cells.**

|  |  | **k_off_ (s^-1^)** |
| --- | --- | --- |
| **Wuhan-1** | **Engineered spike** | 2.2 x 10^-4±0.2^ |
|  | **Whole virus** | 2.1 x 10^-4±0.2^ |
